# Light spectrum effects on micropropagation and gene expression of *Bucephalandra* sp. in a temporary immersion system for sustainable production

**DOI:** 10.3389/fpls.2025.1660632

**Published:** 2025-12-02

**Authors:** Chanakan Laksana, Onsulang Sophiphun, Paitoon Kaewhom, Sontichai Chanprame

**Affiliations:** 1 Faculty of Agricultural Technology, Burapha University, Sakaeo Campus, Sakaeo, Thailand; 2 Department of Agronomy, Faculty of Agriculture at Kamphaeng Saen, Kasetsart University, Nakhon Pathom, Thailand

**Keywords:** aquatic plant, *in vitro* propagation, liquid medium, light regime, photosynthesis efficiency, temporary immersion system, *Bucephalandra*, sustainable production

## Abstract

*Bucephalandra* sp. ‘Wavy Dark Green’ is a commercially valuable ornamental aquatic plant with limited propagation potential due to low multiplication rates and acclimatization challenges. This study aimed to developed an integrated tissue culture protocol by optimizing disinfection procedures, basal media and plant growth regulator, temporary immersion system (TIS) conditions, light quality, and rooting and acclimatization methods. Surface sterilization using 0.1–0.2% HgCl_2_ or a two-step 1.2% NaOCl protocol significantly improved explant survival and reduced contamination. MS medium supplemented with 5 mg/L benzylaminopurine (BA) produced the highest shoot proliferation on solid culture media. In TIS, six immersions per day for 2–3 minutes yielded optimal shoot multiplication and biomass. Blue:red (70:30) and pure blue light enhanced chlorophyll content, photosynthetic efficiency (QY), stomatal opening, and upregulated key light-responsive genes (*psaA*, *psbB*, *petG*, *pskB*, *atpA*, *petA*). Rooting was most effective in ¼ or full-strength MS medium with 0.3 mg/L naphthaleneacetic acid (NAA). Acclimatized plantlets grown in sand exhibited the highest survival and growth. This integrated protocol supports efficient micropropagation and *ex vitro* establishment of *Bucephalandra* sp. ‘Wavy Dark Green’, facilitating its conservation, ornamental use, and sustainable export, in line with SDG 12 (Responsible Consumption and Production).

## 1 Introduction

*Bucephalandra* sp. is a common aquatic plant. Growing aquatic plants such as *Bucephalandra* sp. ‘Wavy Dark Green’ is often used for aquarium design. Recently, there has been an increasing demand for these plants. The increasing global demand for ornamental fish has significantly increased interest in aquatic plants, as enthusiasts seek to create natural and aesthetically appealing environments for their aquatic pets (Grand View Research, 2023; IMARC Group, 2024). This trend is particularly pronounced in international markets such as Europe, Asia, and the United States. Aquatic plants are also widely used to enhance gardens and indoor aesthetics (Bai and Bai, 2024). The rising popularity of indoor plant decoration and the desire for green spaces have fueled the expansion of the aquatic plant industry, with several species becoming valuable commodities for export (Cai and Leung, 2020; Thammasiri et al., 2025). ​In freshwater aquariums, several plant genera are highly popular due to their aesthetic appeal and ease of care. Notable examples include *Anubias*, *Cryptocoryne*, *Hygrophila*, *Vallisneria*, and *Echinodorus*. Among these, *Bucephalandra*, a slow-growing genus endemic to Borneo, has attracted significant attention due to its diverse leaf morphology, adaptability, and ornamental value. Wong and Boyce (2014) described 19 new species of *Bucephalandra*, highlighting the remarkable diversity of the genus. These small Araceae plants can grow attached to hard substrates such as rocks and driftwood, making them especially suitable for aquascaping. However, the extremely slow natural growth of the genus has led to overharvesting as its popularity rises, posing threats to wild populations (Biotope, 2018). This underscores the urgent need for efficient *ex situ* propagation strategies for both commercial and conservation purposes.

Plant tissue culture, or micropropagation, offers a powerful method for propagating rare and slow-growing aquatic species. *In vitro* culture enables year-round production of uniform, disease-free plantlets and supports conservation by overcoming limitations associated with conventional propagation methods, such as rhizome division and low germination rates (Bamane et al., 2023). Moreover, micropropagation eliminates pathogens and algae commonly found in wild specimens, ensuring high-quality plant production (Mata-Rosas et al., 2010). Once an optimal culture medium is established, the next challenge involves scaling up plantlet production through more efficient systems. Numerous studies have reported successful micropropagation of ornamental aquatic plants using various explants, such as the rhizomes or shoot tips of *Anubias barteri* (Baby et al., 2024; Huang et al., 1994). Kanchanapoom et al. (2012) focused on shoot tip culture and ploidy stability, whereas Boonmee et al. (2024) optimized shoot induction in *Anubias* sp. ‘White’ using adenine sulfate and BAP. Similarly, Barpete et al. (2019) investigated the effect of gibberellic acid on *in vitro* flowering in *A. barteri*. For *Cryptocoryne*, Klaocheed et al. (2018) and Stanly et al. (2010) developed protocols for *C. wendtii* and *C. beckettii*, and Pongchawee et al. (2007) refined protoplast culture and direct organogenesis techniques in *C. wendtii*. Due to increasing concerns over overharvesting, *Bucephalandra* species have also received attention. Laohavisuti et al. (2017) studied the effects of disinfectants and growth regulators, and Septianingsih et al. (2024) examined the use of BA and coconut water to promote shoot proliferation. These studies collectively highlight the potential of *in vitro* culture as a scalable and sustainable approach to aquatic plant propagation.

Recent advancements in tissue culture include the use of bioreactor systems, which enhance automation, propagation rates, and labor efficiency. Bioreactors have been shown to increase biomass accumulation, shoot multiplication, and physiological responses in various plant species (Alvard et al., 1993; Murthy et al., 2023; Sivakumar et al., 2005). The temporary immersion system (TIS) is particularly effective, providing superior gas exchange and nutrient availability while minimizing hyperhydricity and contamination (Etienne and Berthouly, 2002; Georgiev et al., 2014). TIS has proven beneficial in aquatic plants by improving multiplication rates and morphological quality. In Thailand, Teanchartsakul et al. (2019) reported that *Anubias barteri* ‘Broad Leaf’ cultured using TIS at six 5-minute immersions per day showed significantly better shoot proliferation than cultures on semi-solid media. Similarly, *Echinodorus aflame* has been propagated via TIS under national innovation programs (Chamnanwech, 2021). Georgiev et al. (2014) also successfully applied TIS to aquatic plants such as *Bacopa monnieri* and *Hygrophila* spp.

Light quality is another critical factor influencing plant development during *in vitro* culture. The advent of LED technology has enabled the delivery of precise light spectra that enhance photosynthesis and morphogenesis (Gupta and Jatothu, 2013). Research has shown that specific red:blue light ratios can improve shoot multiplication and various physiological traits (Cavallaro et al., 2022; Costa et al., 2021; Ptak et al., 2024). Another essential element in micropropagation is the composition of the culture medium, which must be optimized for each species in terms of macronutrients, micronutrients, vitamins, and plant growth regulators (Murashige and Skoog, 1962; George et al., 2008).

For *Bucephalandra* sp., optimal *in vitro* conditions are not yet fully established. Propagation of this plant through tissue culture often encounters problems at various stages because the plant is small, grows in environments prone to microbial contamination, and has delicate tissues. Surface sterilization to obtain aseptic explants for tissue culture is difficult. Moreover, this plant exhibits low growth and multiplication rates. Culturing with the appropriate medium composition, type, and concentration of growth regulators can help overcome these issues. In addition, the survival rate of plants after transplantation to natural conditions is the ultimate factor indicating the success of propagating this plant via tissue culture. This study aimed to improve the micropropagation procedure for this aquatic plant by systematically assessing key factors. These included evaluating sterilization procedures, optimizing media formulations, and determining suitable hormone types and concentrations to promote shoot induction and overall plant growth, as well as establishing the appropriate acclimatization protocols to maximize the survival rate. Because LEDs have been shown to benefit *in vitro* plant growth, the effects of LED lighting on the plant’s growth, multiplication rates, expression of light-responsive genes, and key physiological traits. were also investigated. These comprehensive findings will contribute to the development of a scalable and sustainable tissue culture system for *Bucephalandra* sp., supporting both commercial production and species conservation.

## 2 Materials and methods

### 2.1 Plant material and explant sterilization

The experiments were conducted from October 2023 to December 2024 at the Plant Tissue Culture and Molecular Biology Laboratory, Faculty of Agricultural Technology, Burapha University Sakaeo Campus. *In vitro* cultures of *Bucephalandra* sp. ‘Wavy Dark Green’ were initiated from apical meristem buds collected from healthy, disease-free plants. The apical buds were first washed thoroughly under running tap water to remove surface debris. Sterilization was performed under aseptic conditions within a laminar flow cabinet. Surface sterilization involved various treatments using sodium hypochlorite (NaOCl) and mercuric chloride (HgCl_2_), either individually or in combination, as detailed below:

Treatments 1–4: Explants were immersed in 0.6% NaOCl for 5, 10, 15, or 20 minutes.
Treatments 5–8: Explants were immersed in 1.2% NaOCl for 5, 10, 15, or 20 minutes.
Treatments 9–11: Explants were first treated with 1.2% NaOCl for 5 or 10 minutes, followed by a second sterilization step using 0.6% NaOCl for either 5 or 10 minutes.
Treatments 12–13: Explants were treated with 0.6% NaOCl for 5 minutes, followed by a second treatment with either 0.05% or 0.1% HgCl_2_ for 5 minutes.
Treatments 14–16: Explants were immersed in 0.05%, 0.1%, or 0.2% HgCl_2_ alone for 10 minutes.

All treatments were preceded by an initial rinse in 70% ethanol for 1 minute. The commercial bleach used as the NaOCl source contained 6.0% available chlorine. After sterilization, explants were rinsed three times with sterile distilled water, each rinse lasting 1 minute. The sterilized explants were then transferred to Murashige and Skoog (MS) medium supplemented with 0.2 mg/L benzylaminopurine (BA), 30 g/L sucrose, and 8 g/L agar, with the pH adjusted to 5.8. Cultures were maintained at 25/23 °C under a 16/8-hour light/dark photoperiod using fluorescent light at an intensity of 36 μmol m^-2^ s^-1^. The number and percentage of surviving and contamination-free explants were recorded at two-day intervals. Explants obtained from the meristems after four weeks of cultivation were used for subsequent multiplication experiments.

### 2.2 Influence of benzylaminopurine and naphthaleneacetic acid on multiple shoot formation in *Bucephalandra* sp. ‘Wavy Dark Green’

Sterile explants of *Bucephalandra* sp. ‘Wavy Dark Green’ were aseptically cultured on MS hormone-free medium containing 30 g/L sucrose for 30 days to eliminate residue BA in the explants prior to transfer to multiple shoot induction media. The media consisted of MS salts supplemented with various concentrations of benzylaminopurine (BA) at 0, 1, 2, 3, 4, and 5 mg/L, in combination with naphthaleneacetic acid (NAA) at concentrations of 0, 0.1, 0.3, and 0.5 mg/L. The pH of the medium was adjusted to 5.7, and 8 g/L agar was used as a solidifying agent. Media were sterilized by autoclaving at 121 °C for 15–20 minutes. Each treatment was replicated three times, with five explants per replicate. All cultures were maintained at 25 ± 2 °C under a 16-hour photoperiod, using 40 W white fluorescent tubes at a light intensity of 30 μmol m^-2^ s^-1^. Observations were made weekly over a 45-day period. Data collected included the number of multiple shoots per explant, average shoot length (cm), number of leaves, number of roots, and root length (cm).

### 2.3 Root induction

After multiple shoot formation, individual shoots were transferred to full-strength, half-strength, or quarter-strength MS medium, as well as MS supplemented with 0.1–0.3 mg/L NAA, for adventitious root induction. Cultures were maintained under a 16-hour light/8-hour dark photoperiod at 25 ± 2 °C, using 40 W white fluorescent tubes providing 30 μmol m^-2^ s^-1^ light intensity. Each treatment was replicated three times, with ten shoots per replicate. After four weeks of cultivation, the number of roots, root length (cm), and rooting rate (%) were recorded.

### 2.4 Acclimatization

Following successful *in vitro* multiplication and rooting, healthy and well-developed plantlets were selected. Roots were gently rinsed under running water to remove residual medium. Four different substrates—sand, perlite, vermiculite, and black rice husk—were tested for acclimatization. Plantlets were placed in trays with lids containing small ventilation holes and maintained for two months in a humidity chamber (80–90% relative humidity) to prevent desiccation. Hoagland’s nutrient solution (Hoagland and Arnon, 1950) was applied weekly. The plants were grown under low light conditions (approximately 60% shade) at temperatures ranging from 28–34 °C. After two months, the number of surviving plants and survival rate (%) were recorded.

### 2.5 Effect of frequency and duration in a temporary immersion bioreactor system on multiple shoot formation in *Bucephalandra* sp. ‘Wavy Dark Green’

The effect of immersion frequency and duration on the growth of *Bucephalandra* sp. ‘Wavy Dark Green’ was investigated using a temporary immersion system (TIS) bioreactor. The optimized nutrient medium from previous experiments was used. Five sterilized plantlets were placed into each bioreactor unit containing the prepared liquid culture medium. In the first experiment, immersion frequencies of 3, 4, 5, and 6 times per day (each with a 6-minute duration) were tested. In the second experiment, using the optimal immersion frequency determined from the first, immersion durations of 2, 3, 4, and 5 minutes were evaluated. All cultures were maintained at 25 ± 2 °C under a 16-hour photoperiod with a light intensity of 30 μmol m^-2^ s^-1^, using 40 W cool white fluorescent tubes. Each treatment was replicated three times, with five explants per replicate. After four weeks of culturing, data were recorded on the number of multiple shoots per explant and average shoot length (cm).

### 2.6 Evaluation of light quality on plant growth and multiplication rate in a TIS

Each plant species responds differently to light types (wavelength ranges), which directly affects the development and growth of that species under tissue culture conditions. Therefore, *Bucephalandra* sp. ‘Wavy Dark Green’ plants were cultured in a TIS under LED light sources, which allow precise control of wavelength and wavelength ratios (light color types) compared to fluorescent lighting. Five plantlets were cultured in each bioreactor unit containing MS liquid medium supplemented with 5 mg/L BA. Based on prior optimization, immersion was set at six times per day, each lasting two minutes.

**Figure 1:**
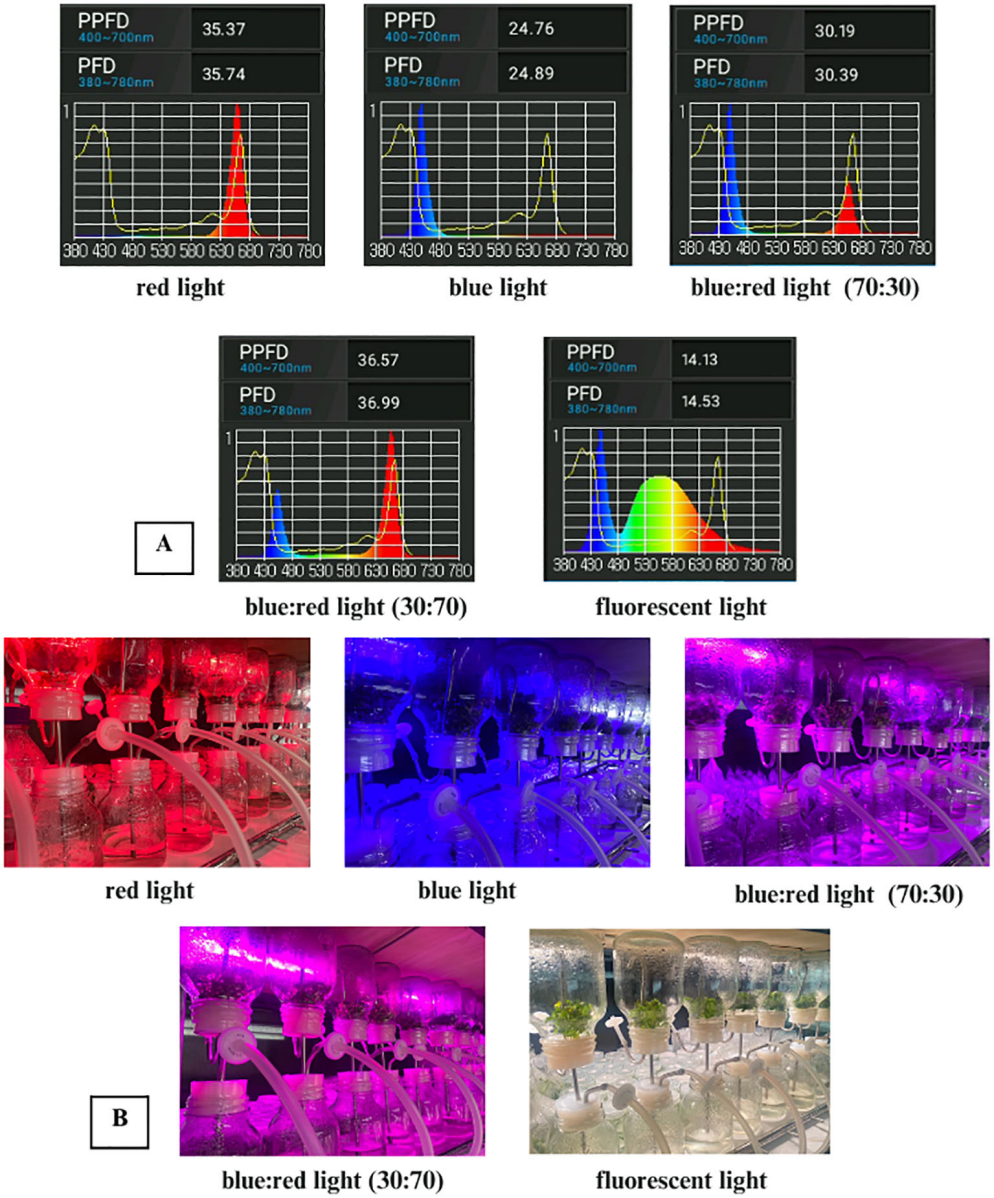
**(A)** Spectral light distribution of red, blue, blue:red (70:30 and 30:70), and white fluorescent light. **(B)***In vitro* culture of *Bucephalandra* sp. ‘Wavy Dark Green’ in a temporary immersion bioreactor system (TIS) under different light quality treatments: red, blue, blue:red (70:30), blue:red (30:70), and white fluorescent light.

Light treatments included white fluorescent light, pure blue and red light, a 70:30 blue:red ratio, and a 30:70 blue:red ratio. Spectral distribution, photosynthetic photon flux density (PPFD), and photon flux density (PFD) values for each treatment were measured using an LI-180 spectrometer (LI-COR, USA) and are presented in **Table 1**; **Figure 1**. All cultures were maintained at 25 ± 2 °C under a 16-hour photoperiod at a light intensity of 30 μmol m^-2^ s^-1^. Each treatment included three replicates, with five explants per replicate. After four weeks, data were collected on percentage change in fresh weight, number of multiple shoots per explant, average shoot length (cm), number of leaves, number of roots, root length (cm), and stomatal number and length.

**Table 1:** PPFD and PFD analysis as measured using a LI-180 spectrometer lighting analyzer color photometer.

Light	PPFD (400–700 nm)	PFD (380–780 nm)
Red	35.37	35.74
Blue	24.76	24.89
70:30 Blue and Red Ratio	30.19	30.39
30:70 Blue and Red Ratio	36.57	36.99
Fluorescent Light	14.13	14.53

### 2.7 RNA isolation and quantitative real-time PCR

Quantitative real-time PCR (qRT-PCR) was conducted to assess the expression patterns of six genes in *Bucephalandra* sp. ‘Wavy Dark Green’ grown under different light conditions. After four weeks of culture, total RNA was extracted from fresh leaves of each light treatment group following the protocol described by Laksana and Chanprame (2015). Reverse transcription was performed using the SuperScript™ VILO™ cDNA Synthesis Kit (Invitrogen, USA). Gene expression levels were normalized against the *actin* reference gene to ensure accurate quantification. Gene-specific primers for *psaA*, *psbB*, *pskB*, *petA*, *petG*, and *atpA* were designed based on sequence alignments from Araceae species using MEGA11 software (**Table 2**). qRT-PCR reactions were performed in a final volume of 20 µL, containing 100 ng of cDNA template, 1× SensiFAST SYBR^®^ No-ROX mix (Bioline Reagents Ltd.), and 0.4 µM of each primer. Amplification was carried out on a CFX96 Touch™ Real-Time PCR Detection System (Bio-Rad^®^, USA) using the following cycling conditions: initial denaturation at 95 °C for 30 seconds, followed by 40 cycles of denaturation at 94 °C for 5 seconds, annealing at 58 °C for 15 seconds, and extension at 72 °C for 10 seconds. Each sample included three biological replicates and three technical replicates. Gene expression levels were calculated using the 2^–ΔΔCq method (Livak and Schmittgen, 2001).

**Table 2:** Specific primer pairs for quantitative real-time PCR analysis of *Bucepharandra* sp. Wavy Dark Green growth under different light conditions.

Group of gene	Name of gene	Sequence
Subunit of photosystem I	*psaA*	CGGGGTTACTAGGACTTGGG ACTGGGATAAAGCTGAGCCA
Subunit of photosystem II	*psbB*	GCATTGGGTGTATTGGGACC CGGACACCCATATTCCAGGA
	*psbK*	TTCTGCCCTTTCTTCAAGCA AAGTACAGGTATGACGGGCA
Subunits of cytochrome	*petA*	GGAAACAGAGGAAGGGGTCA ACAAGAAGTTCCGGTCCTGG
	*petG*	ATGATTGAAGTTTTTCTA TCAAAGGTCCAGCTGATC
Subunits of ATP synthase	*atpA*	CTCACGGTGGAAGAGCAGAT TCGGTGAATGTCTTGGTGGA

### 2.8 Photosynthesis efficiency of *Bucephalandra* sp. ‘Wavy Dark Green’ cultured in a TIS and under LED lighting

To investigate the influence of the TIS culture system combined with different LED light spectra, several physiological parameters related to photosynthetic efficiency were measured to assess the plant’s responses to these culture conditions.

#### 2.8.1 Chlorophyll and carotenoid content

Chlorophyll (a, b, and total) and carotenoid (β-carotene) contents were extracted following the method of Kulkarni and Nikolov (2018), with minor modifications. For each light treatment, 0.2 g of fresh leaf tissue was placed in a vial and extracted with 10 mL of a solvent mixture of acetone and ethanol (2:1, v/v). Samples were incubated in darkness at room temperature for 48 h. Absorbance was measured at 663 nm (chlorophyll a), 645 nm (chlorophyll b), and 470 nm (carotenoids) using a UV-Vis spectrophotometer (T60 UV-Visible Spectrophotometer, PG Instruments Ltd., UK). Each analysis was performed in triplicate.

#### 2.8.2 Anthocyanin content

Anthocyanin content was determined from 0.2 g of fresh leaves collected from each light condition. The tissue was ground in 10 mL of a methanol:water:HCl solution (16:3:1, v/v/v) and incubated in darkness at room temperature for 48 h. After incubation, samples were centrifuged at 13,000 rpm for 10 minutes. The supernatant was collected, and absorbance was recorded at 530 nm and 653 nm using the same spectrophotometer. All measurements were performed in triplicate.

#### 2.8.3 Chlorophyll fluorescence measurement and analysis

To evaluate photosynthetic efficiency, chlorophyll fluorescence parameters were measured using the FluorPen FP 110 (Photon Systems Instruments), a portable pulse-amplitude modulated (PAM) fluorometer. Prior to measurements, leaves from plants exposed to different light conditions were dark-adapted using standard leaf clips to fully open all photosystem II (PSII) reaction centers. The parameters measured included Instantaneous Chlorophyll Fluorescence (Ft), Quantum Yield (QY), and Non-Photochemical Quenching (NPQ). Ft reflected baseline fluorescence, QY indicated the maximum photochemical efficiency of PSII, and NPQ assessed heat dissipation efficiency. All readings were taken in triplicate.

#### 2.8.4 Stomatal density, size, and aperture measurement

Stomatal characteristics were assessed using the clear nail polish impression method. A thin layer of transparent nail polish was applied to the abaxial surface of fully expanded leaves and allowed to dry. The dried film was gently peeled off using transparent adhesive tape and mounted on microscope slides. Observations were made using a Zeiss Primo Star compound light microscope. Stomatal density was determined at 100× total magnification (10× objective lens) by counting the number of stomata within a defined field of view and expressed as the number of stomata per square millimeter (mm²), based on calibrated field dimensions. Stomatal size and aperture were assessed at 400× magnification using an ocular micrometer. Stomatal length (L) was measured as the distance between the ends of the guard cells, and stomatal width (W) as the maximum width perpendicular to the longitudinal axis. At least 30 stomata were measured per sample (n = 30).

### 2.9 Statistical analysis

All data were analyzed using the R programming environment (R Core Team, 2012). One-way analysis of variance (ANOVA) was used to determine the significance of treatment effects. Differences among means were assessed using Tukey’s HSD test at a significance level of *p* < 0.05.

## 3 Result

**Figure 2:**
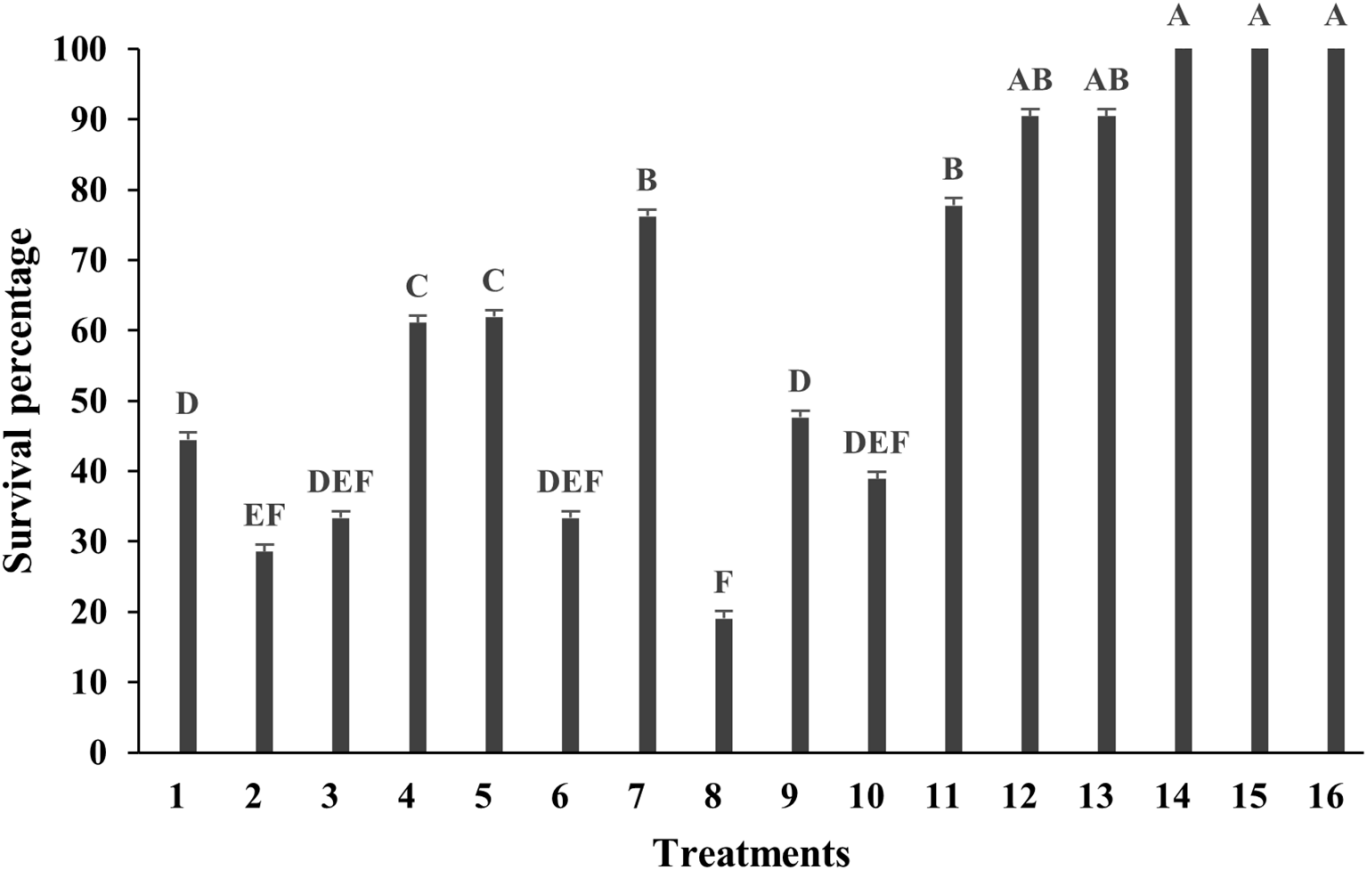
Survival percentage of *Bucephalandra* sp. ‘Wavy Dark Green’ after surface sterilization with different treatments using sodium hypochlorite (NaOCl) and mercuric chloride (HgCl_2_). Treatments T1–T8 represent single-stage sterilization with various concentrations and durations of NaOCl; T9–T11 are two-step sterilization treatments; T12–T16 involve combinations or single-stage treatment with HgCl_2_. Bars indicate survival percentages after contamination assessment. Sterilization treatments were as follows: T1–T4: 0.6% NaOCl for 5, 10, 15, and 20 mins, respectively, T5–T8: 1.2% NaOCl for 5, 10, 15, and 20 mins, respectively, T9: 1.2% NaOCl (5 mins) and 0.6% NaOCl (5 mins), T10: 1.2% NaOCl (10 mins) and 0.6% NaOCl (5 mins), T11: 1.2% NaOCl (10 mins) and 0.6% NaOCl (10 mins), T12: 0.6% NaOCl (5 mins) and 0.05% HgCl_2_ (5 mins), T13: 0.6% NaOCl (5 mins) and 0.1% HgCl_2_ (5 mins) T14–T16: 0.05%, 0.1%, and 0.2% HgCl_2_ for 10 mins, respectively. Different letters above the bars indicate statistically significant differences between treatments (p < 0.05) based on one-way ANOVA followed by Tukey’s HSD test.

**Figure 3:**
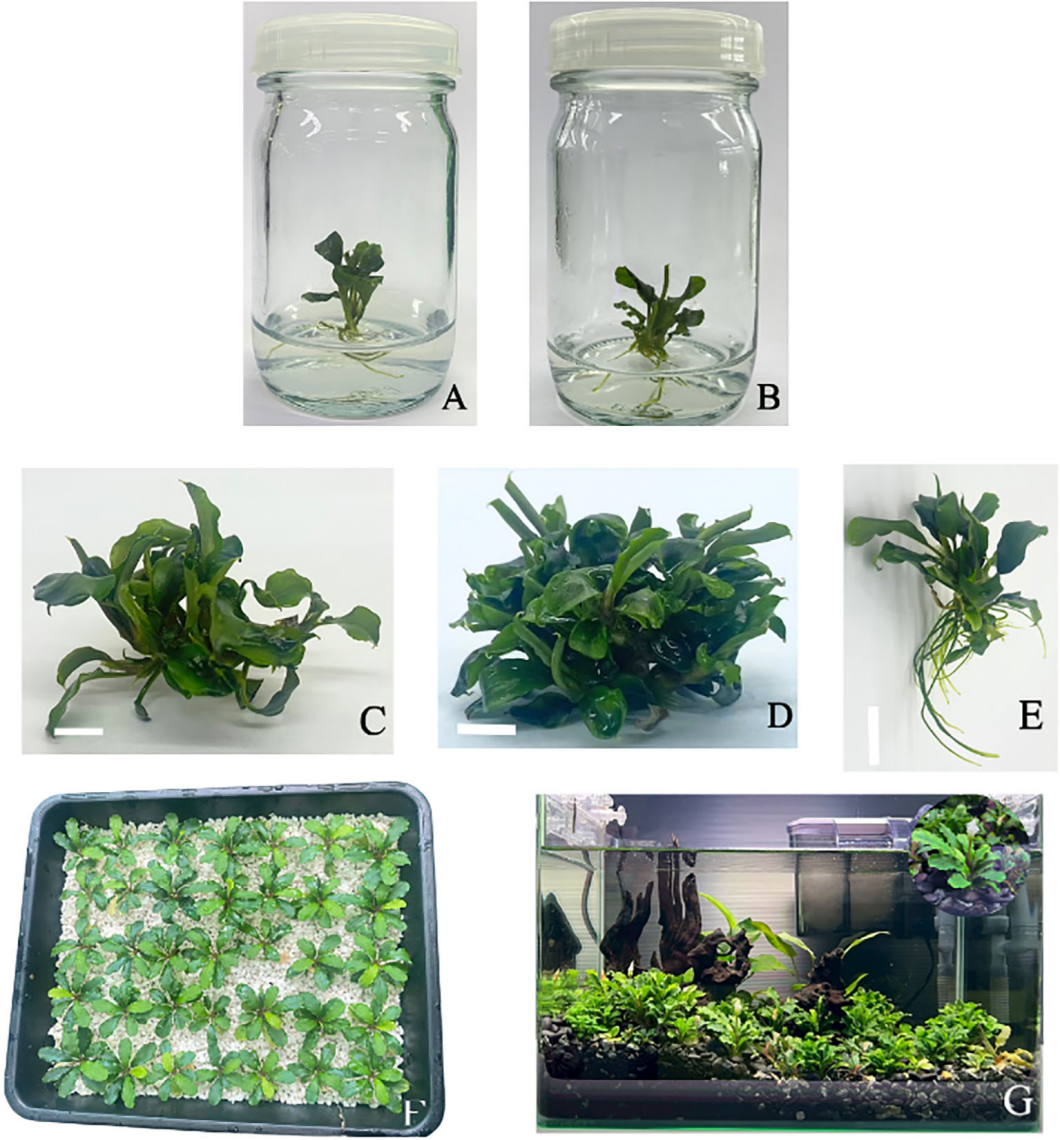
**(A-G)***In vitro* propagation and acclimatization process of *Bucephalandra* sp. ‘Wavy Dark Green’. **(A)** Survived plant after surface sterilization with 0.05% HgCl_2_ for 10 minutes. **(B)** Multiple shoot formation on MS medium supplemented with 5 mg/L BA. **(C)** Growth response in temporary immersion system (TIS) with immersion frequency of 6 times per day. **(D)** Growth response in TIS with immersion duration of 2 minutes and frequency of 6 times per day under blue:red 70:30 treatment. **(E)** Root induction in ¼-strength MS medium without additional auxins, showing the highest root number (~7 roots per explant). **(F)** Acclimatization in perite substrate to provide optimal aeration and drainage conditions. **(G)** Acclimatized plants grown in a display aquarium for ornamental purposes following *in vitro* propagation and *ex vitro* acclimatization.

The effectiveness of various surface sterilization treatments on shoot tips of *Bucephalandra* sp. ‘Wavy Dark Green’ was evaluated using different concentrations and exposure times of sodium hypochlorite (NaOCl) and mercuric chloride (HgCl_2_). Treatments with 0.6% and 1.2% NaOCl alone resulted in survival rates ranging from 14.29% to 71.43%. Moderate efficacy was observed with 1.2% NaOCl for 15 minutes (71.43%, T7) and 0.6% NaOCl for 20 minutes (66.67%, T4). The addition of a second sterilization step generally enhanced decontamination efficiency. For instance, treatment with 0.6% NaOCl for 5 minutes followed by either 0.05% or 0.1% HgCl_2_ resulted in the highest survival rate of 85.71% (T12 and T13) (**Figure 2**). Notably, treatments with HgCl_2_ alone at concentrations of 0.05–0.2% for 10 minutes consistently achieved 100% survival (T14–T16) with no signs of contamination (**Figures 2**, **3A**), demonstrating the superior sterilization efficacy of HgCl_2_.

### 3.1 Influence of BA and NAA on multiple shoot formation in *Bucephalandra* sp. ‘Wavy Dark Green’

The effects of varying concentrations of BA (0–5 mg/L) and NAA (0–0.5 mg/L) on *in vitro* shoot induction and growth performance of *Bucephalandra* sp. ‘Wavy Dark Green’ were evaluated. The results revealed that shoot proliferation was significantly influenced by BA concentration. The highest number of shoots (4.6 ± 0.84) was obtained on MS medium supplemented with 5 mg/L BA without NAA (**Figure 3B**), followed by 4.2 ± 0.55 shoots in media containing either 3 or 4 mg/L BA without NAA. In contrast, the lowest shoot number (1.0 ± 0.00) was recorded in the treatment with 0.3 mg/L NAA alone, highlighting the essential role of cytokinins in shoot induction for this species. The number of leaves per explant varied only slightly across treatments, ranging from 2.4 to 3.2, with no statistically significant differences observed in most cases (**Table 3**). In terms of shoot length, the tallest shoots (1.94 ± 0.13 cm) were recorded in the treatment combining 5 mg/L BA with 0.3 mg/L NAA.

**Table 3:** Effect of various concentrations of benzylaminopurine (BA) and naphthaleneacetic acid (NAA) on multiple shoot formation and root development in *Bucephalandra* sp. ‘Wavy Dark Green’ cultured in MS medium for four weeks.

No.	MS medium supplemented with		Number of shoots	Number of leaves	Height (cm)	Number of roots	Length of roots (cm)
	BA	NAA					
1	0	0	1.2 ± 0.84fg	2.80 ± 0.21ab	1.66 ± 0.22abcd	1.40 ± 0.70abcd	0.50 ± 0.71bc
2	0	0.1	1.4 ± 0.00efg	1.20 ± 0.37cd	1.26 ± 0.43de	0.00d	0.00d
3	0	0.3	1.0 ± 0.00g	0.80 ± 0.27d	1.00 ± 0.27e	0.00d	0.00d
4	0	0.5	1.2 ± 0.00fg	2.40 ± 0.38ab	1.52 ± 0.45abcd	2.00 ± 0.44abcd	0.2 ± 0.00cd
5	1	0	2.2 ± 0.00bcdef	2.60 ± 0.41ab	1.56 ± 0.41abcd	0.00d	0.00d
6	1	0.1	1.8 ± 0.00defg	2.60 ± 0.43ab	1.48 ± 0.00abcd	0.00d	0.00d
7	1	0.3	1.6 ± 0.00defg	2.80 ± 0.43ab	1.68 ± 0.43abcd	2.00 ± 0.08abcd	0.32 ± 0.09cd
8	1	0.5	2.0 ± 0.00cdefg	2.80 ± 0.45ab	1.32 ± 0.45cde	2.20 ± 0.00abc	0.36 ± 0.18cd
9	2	0	3.0 ± 0.00bc	3.20 ± 0.00a	1.38 ± 0.00bcde	0.00d	0.00d
10	2	0.1	2.6 ± 0.45bcd	2.60 ± 0.00ab	1.58 ± 0.55abcd	0.60 ± 0.54cd	0.20 ± 0.17cd
11	2	0.3	1.2 ± 0.45fg	2.40 ± 0.00ab	1.56 ± 0.00abcd	1.60 ± 0.28abcd	0.36 ± 0.11cd
12	2	0.5	1.0 ± 0.45g	2.60 ± 0.45ab	1.32 ± 0.50cde	2.80 ± 0.17abc	0.26 ± 0.16cd
13	3	0	4.2 ± 0.55a	2.80 ± 0.45ab	1.66 ± 0.45abcd	0.00d	0.00d
14	3	0.1	1.4 ± 0.55efg	2.80 ± 0.55ab	1.52 ± 0.36abcd	1.40 ± 0.26abcd	0.60 ± 0.18bc
15	3	0.3	2.6 ± 0.45bcd	2.80 ± 0.50ab	1.48 ± 0.50abcd	3.00 ± 0.18ab	0.34 ± 0.18cd
16	3	0.5	1.2 ± 0.45fg	1.80 ± 0.50bc	1.42 ± 0.30bcde	1.60 ± 0.15abcd	0.56 ± 0.09bc
17	4	0	4.2 ± 0.55a	3.20 ± 0.43a	1.76 ± 0.43abc	2.40 ± 0.13abc	1.06 ± 0.13a
18	4	0.1	2.4 ± 0.84bcde	3.00 ± 0.36a	1.84 ± 0.18ab	3.20 ± 0.12ab	0.84 ± 0.00ab
19	4	0.3	1.2 ± 0.45fg	3.00 ± 0.37ab	1.68 ± 0.30abcd	1.20 ± 0.14bcd	0.24 ± 0.14cd
20	4	0.5	2.4 ± 0.45bdce	3.00 ± 0.38a	1.58 ± 0.22abcd	2.40 ± 0.08abc	0.54 ± 0.08bc
21	5	0	4.6 ± 0.84a	3.00 ± 0.39a	1.78 ± 0.15abc	3.60 ± 0.16a	1.1 ± 0.12a
22	5	0.1	3.2 ± 0.89b	3.00 ± 0.40a	1.74 ± 0.24abc	2.40 ± 0.34abc	0.46 ± 0.08bc
23	5	0.3	1.6 ± 0.55defg	3.00 ± 0.41a	1.94 ± 0.13a	3.20 ± 0.08ab	0.64 ± 0.09bc
24	5	0.5	2.0 ± 0.84cdefg	3.00 ± 0.42ab	1.22 ± 0.31de	0.00d	0.00d

Each value is presented as mean ± standard deviation (n = 10).

Different lowercase letters within each column indicate statistically significant differences among treatments at p < 0.05 as determined by one-way ANOVA followed by Tukey’s HSD test.

Root development was notably influenced by the presence of NAA. The greatest number of roots (3.6 ± 0.16) was observed in explants cultured on medium containing 5 mg/L BA without NAA, whereas many treatments with BA alone did not support root formation. The longest roots (1.10 ± 0.12 cm) were also observed in this treatment, followed closely by the 4 mg/L BA without NAA treatment (1.06 ± 0.13 cm) (**Table 3**). These results indicate that while higher concentrations of BA effectively promote shoot proliferation, low levels of NAA can moderately enhance root elongation. However, treatments with auxin alone inhibited both shoot and root formation. Overall, the combination of 5 mg/L BA without NAA was the most effective condition for inducing multiple shoots and promoting root elongation in *Bucephalandra* sp. ‘Wavy Dark Green’.

### 3.2 Root induction

**Figure 4:**
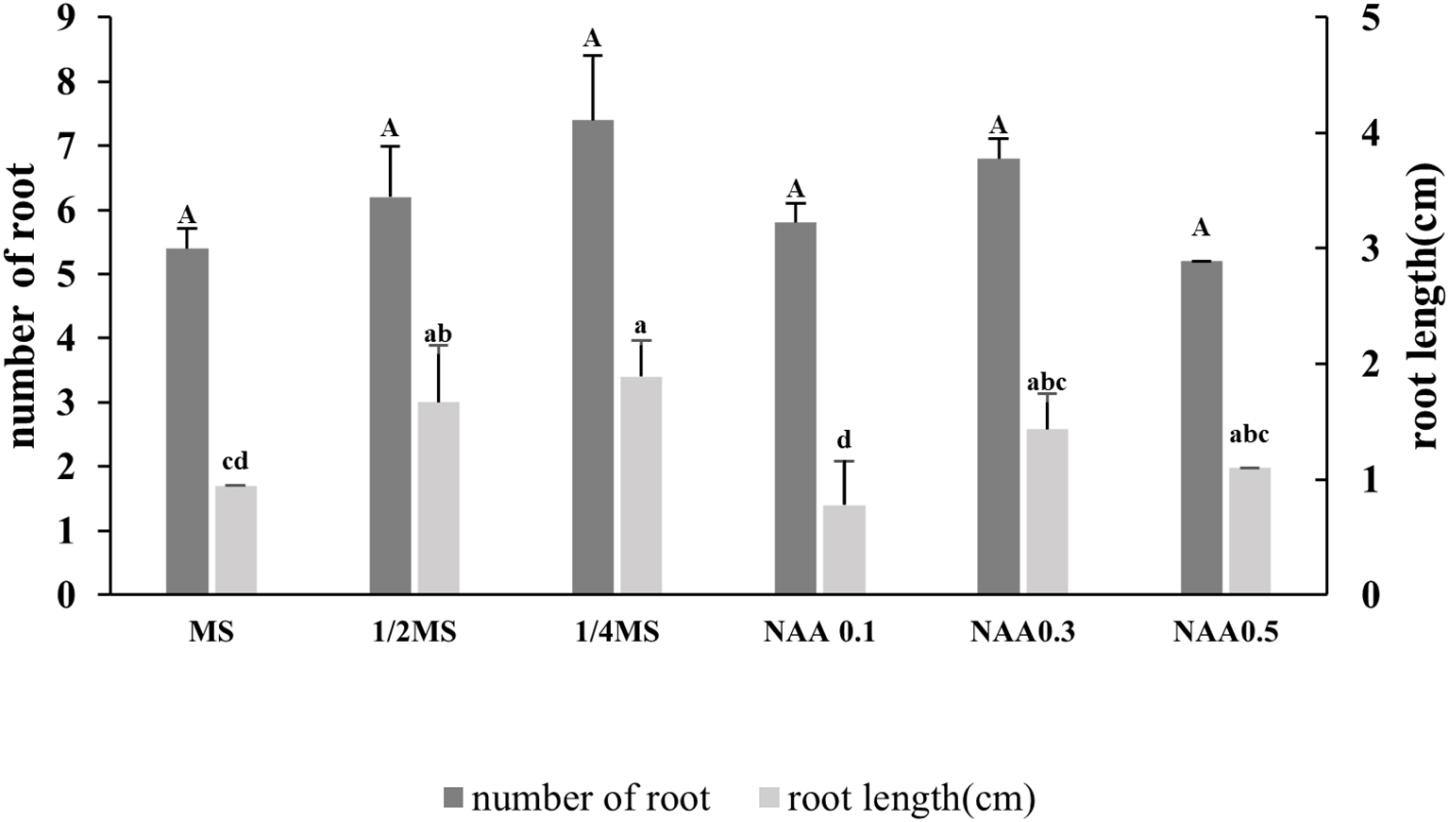
Effect of different nutrient media and NAA concentrations on root induction in *Bucephalandra* sp. ‘Wavy Dark Green’ cultured *in vitro*. Dark gray bars represent the average number of roots per explant, and light gray bars represent average root length (cm). Treatments included full-strength, half-strength, and quarter-strength MS media, as well as MS supplemented with various concentrations of NAA; 0.1, 0.3, and 0.5 mg/L. Different lowercase letters indicate significant differences in root length, and uppercase letters denote differences in root number, based on one-way ANOVA followed by Tukey’s HSD test (p < 0.05). Values are expressed as means ± standard deviations (n = 10).

Root induction in *Bucephalandra* sp. ‘Wavy Dark Green’ was significantly influenced by the type of basal medium and the presence or absence of NAA supplementation. Among the treatments tested, quarter-strength MS medium (¼ MS) without exogenous auxins produced the highest number of roots per explant (7.4 ± 0.89) (**Figures 4**), although the difference was not statistically significant compared to other treatments. The second most effective treatment was full-strength MS supplemented with 0.3 mg/L NAA (6.8 ± 1.8), followed by half-strength MS (6.2 ± 5.4). The application of NAA at concentrations of 0.1 and 0.5 mg/L did not improve rooting efficiency further (**Figure 4**). In terms of root elongation, ¼ MS again demonstrated superior performance, producing the longest roots (approximately 3.7 cm), significantly longer than those observed in full-strength MS without hormones (1.98 cm) and with 0.1 mg/L NAA (1.40 cm) (p < 0.05). Although 0.3 mg/L NAA moderately increased root number, root length remained shorter than that induced by auxin-free ¼ MS, suggesting a potential inhibitory effect of higher NAA concentrations on root elongation despite promoting root initiation. These findings suggest that reduced salt concentration, in the absence of exogenous auxins, is more favorable for root induction and elongation in *Bucephalandra* sp. ‘Wavy Dark Green’ under *in vitro* conditions.

### 3.3 Acclimatization

During the acclimatization stage, well-rooted *Bucephalandra* plantlets derived from *in vitro* culture were transplanted into four different substrates: sand, perlite, black rice husk, and vermiculite. Plantlet performance was assessed based on survival rate, visual growth characteristics, and morphological parameters such as leaf expansion, shoot elongation, and root establishment over a standardized acclimatization period. Among all treatments, plantlets grown in sand, perlite, and vermiculite exhibited the highest survival rates (100%) and showed superior vegetative development (**Supplementary Figure S1**). These plantlets demonstrated more vigorous shoot elongation, greater leaf number and size, and more extensive root systems compared to those cultivated in black rice husk. In contrast, the black rice husk treatment resulted in the poorest outcomes, including reduced survival rates, signs of leaf chlorosis, and limited root development (**Supplementary Figure S1**). These results suggest that sand, perlite, and vermiculite offer optimal physical conditions for root aeration and water drainage, supporting improved physiological adaptation and morphological stability during the critical transition from *in vitro* to *ex vitro* conditions.

### 3.4 Optimization of temporary immersion system conditions for multiple shoot formation in *Bucephalandra* sp. ‘Wavy Dark Green’

The optimization of the TIS utilized MS medium supplemented with 5 mg/L BA in two sequential experiments.

#### 3.4.1 Effect of immersion frequency

The immersion duration was fixed at 6 minutes, while frequency varied. Increasing the immersion frequency from three to six times per day significantly enhanced overall biomass and shoot proliferation. The highest biomass increase (1282.80%) and shoot number (8.33 ± 0.81 shoots/explant) were achieved with six immersions per day (fixed at 6 minutes duration), compared to the lowest biomass (806.89%) and shoots (6.87 ± 0.42) at three immersions (**Table 4**; **Figure 3C**; **Supplementary Figure S2**). Rooting, conversely, was notably suppressed at higher frequencies (**Table 4**).

**Table 4:** Effect of immersion frequency (3, 4, 5, and 6 times per day) in a temporary immersion system (TIS) on multiple shoot formation and root induction in *Bucephalandra* sp. ‘Wavy Dark Green’.

Immersion frequency (time)	Percentage change in weight before and after cultivation	Number of shoots per explants	Average shoot height (cm)	Average number of leave	Average number of roots	Average root length (cm)
3	806.89	6.87 ± 0.42b	2.61 ± 0.40b	3.8 ± 0.35a	0.80 ± 0.06a	0.14 ± 0.16b
4	952.46	7.13 ± 0.31b	2.60 ± 0.14b	3.73 ± 0.23a	0.76 ± 0.06a	0.43 ± 0.19a
5	1126.46	8.00 ± 0.72ab	3.1 ± 0.32ab	3.73 ± 0.12a	0.33 ± 0.02b	0.053 ± 0.09b
6	1282.80	8.33 ± 0.81a	3.49 ± 0.31b	3.67 ± 0.31a	0.20 ± 0.00b	0.10 ± 0.00b

The cultures were maintained on MS medium supplemented with 5 mg/L BA for four weeks. Values represent the mean ± standard deviation (n = 5).

Different lowercase letters within the same column indicate statistically significant differences at p < 0.05 as determined by one-way ANOVA followed by Tukey’s HSD test.

#### 3.4.2 Effect of immersion duration

Following the frequency study, the second experiment fixed the frequency at six times per day and varied the immersion duration (2 to 5 minutes). Biomass accumulation increased linearly with longer durations, peaking at 5 minutes (1358.73% weight gain) (**Table 5**). However, the highest number of shoots (12.2 ± 0.85) and tallest plants (2.23 ± 0.24 cm) were observed at the 2-minute duration (**Table 5**; **Figure 3D**; **Supplementary Figure S3**). Although the differences in shoot number were not statistically significant across durations, plant height at 2 and 4 minutes was significantly greater than at 5 minutes, indicating that excessive immersion may inhibit elongation growth. Rooting was absent at 2 minutes, while the 3-minute duration produced the highest average number of roots (3.87 ± 0.64, **Table 5**). In conclusion, while longer durations enhance total biomass, shorter durations (2 minutes) optimized shoot proliferation and elongation. Considering both efficiency and productivity, the protocol of six immersions per day for 2 minutes is the most effective TIS condition for maximizing high-quality shoot production of *Bucephalandra* sp.

**Table 5:** Effect of immersion duration (2–5 minutes) at a fixed frequency of six times per day in a temporary immersion system (TIS) on multiple shoot formation and root development in *Bucephalandra* sp. ‘Wavy Dark Green’.

Immersion duration (min)	Percentage change in weight before and after cultivation	Average number of shoots	Average shoot height (cm)	Average number of leaves	Average number of roots	Average root length (cm)
2	908.82	12.2 ± 0.85a	2.23 ± 0.24a	3.2 ± 0.35a	0c	0c
3	1001.08	10.4 ± 0.53ab	1.65 ± 0.05ab	3 ± 0.20ab	3.87 ± 0.64a	0.49 ± 0.04ab
4	1186.82	10.33 ± 0.70ab	2.05 ± 0.17a	2.6 ± 0.20b	3.08 ± 0.42b	0.54 ± 0.12a
5	1358.73	10.27 ± 0.53ab	1.72 ± 0.12b	2.8 ± 0.35ab	3.73 ± 0.81a	0.35 ± 0.01b

The cultures were maintained on MS medium supplemented with 5 mg/L BA for four weeks. Values are expressed as means ± standard deviation (n = 5).

Different letters within each column indicate statistically significant differences at p < 0.05 according to one-way ANOVA followed by Tukey’s HSD test.

### 3.5 Effects of light spectrum on growth, gene expression, and photosynthetic efficiency

The spectral composition of light profoundly influenced the growth parameters, photosynthetic pigment accumulation, and gene expression. The blue:red light spectrum at a 70:30 ratio consistently provided the most favorable conditions across all analyzed metrics. Under this optimal light quality, *Bucephalandra* displayed the highest multiplication rate, producing 14.40 ± 0.94 shoots per explant, substantial biomass accumulation (1323.99% increase in fresh weight), and robust leaf development (**Table 6**; **Supplementary Figure S4**). The superior growth correlated strongly with the maximum accumulation of total chlorophyll (63.45 ± 0.97 mg/kg) and carotenoids (63.45 ± 0.97 mg/kg) (**Table 7**).

**Table 6:** Effects of different light qualities on growth parameters of *Bucephalandra* cultured in TIS.

Treatments	Percentage change in weight before and after cultivation	Average number of shoots	Average shoot height (cm)	Average number of leaves/Shoot
Blue	1218.22	11.07c	2.15ab	3.07ab
Red	256.32	6.40e	1.96b	2.67b
Blue: Red (70:30)	1323.99	14.40a	1.90b	2.40b
Blue: Red (30:70)	654.04	8.60d	1.98b	2.40b
fluorescent	1305.32	12.13b	2.46a	3.73a

Different letters within a column indicate statistically significant differences at p < 0.05 based on one-way ANOVA followed by Tukey’s HSD test.

**Table 7:** Influence of light spectra on photosynthetic pigments and anthocyanin levels (mg/kg) in *Bucephalandra* grown in TIS.

Treatments	Chlorophyll		Carotenoid	Anthocyanin
	A	B		
Blue	43.65 ± 1.15d	21.08 ± 1.04d	38.18 ± 1.01d	0.17 ± 0.009a
Red	56.77 ± 1.29c	27.10 ± 0.63c	46.87 ± 1.51c	0.13 ± 0.002c
Blue: Red (70:30)	77.06 ± 0.76a	33.50 ± 0.50a	63.45 ± 0.97a	0.10 ± 0.007d
Blue: Red (30:70)	76.67 ± 0.82a	32.84 ± 0.46b	63.11 ± 0.53a	0.14 ± 0.006b
Fluorescent	70.18 ± 1.27b	26.72 ± 0.68c	59.57 ± 1.01b	0.12 ± 0.006c

Different letters within a column indicate significant differences among treatments at p < 0.05 (ANOVA, Tukey’s HSD).

**Figure 5:**
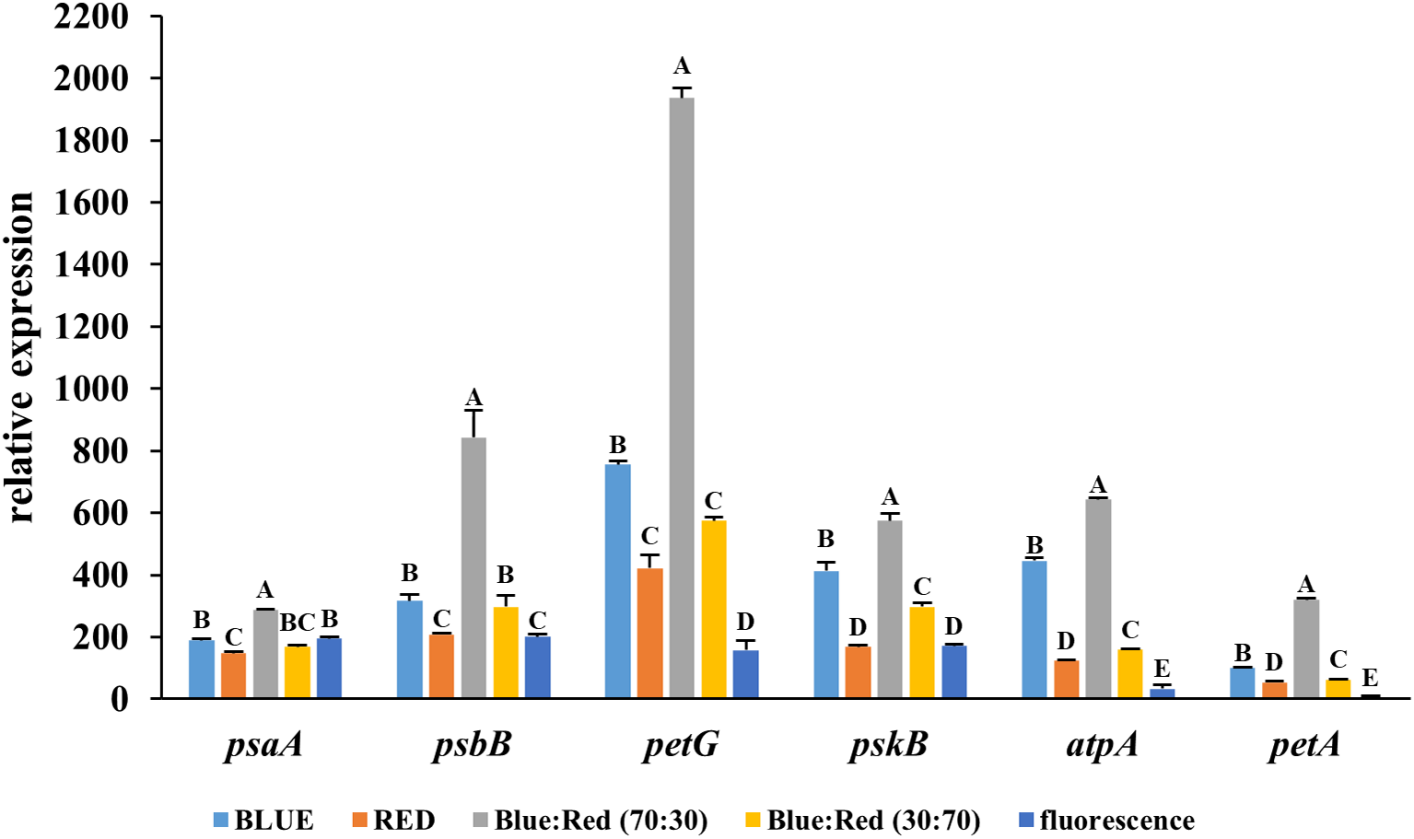
Relative expression levels of light-responsive genes (*psaA*, *psbB*, *petG*, *pskB*, *atpA*, and *petA*) in *Bucephalandra* cultured in a temporary immersion bioreactor (TIS) under six different light conditions: blue, red, blue:red (70:30), blue:red (30:70), fluorescent light. Gene expression was analyzed by quantitative real-time PCR (qRT-PCR), and data were normalized to the reference gene, *actin*. Each bar represents the mean ± standard deviation (n = 3). Different letters above the bars indicate statistically significant differences between treatments (p < 0.05) based on one-way ANOVA followed by Tukey’s HSD test.

The blue:red (70:30) spectrum resulted in the highest expression levels for all six genes examined (*psaA, psbB, petG, pskB, atpA*, and *petA*), indicating the most robust activation of the photosynthetic machinery. Notably, the gene *petG* was strongly upregulated, with its relative expression level exceeding 2000, significantly higher than any other treatment. Similarly, *psbB, pskB*, and *atpA* displayed peak expression, reflecting enhanced activity in photosystem II, the cytochrome *b6f* complex, and ATP synthesis. In contrast, fluorescent light consistently yielded the lowest expression levels across all genes tested (**Figure 5**), suggesting the least stimulatory effect on photosynthesis-related gene networks. These results collectively confirm that the blue:red (70:30) light combination is highly effective in activating key light-responsive genes, suggesting enhanced photosynthetic efficiency during *in vitro* TIS culture.

**Figure 6:**
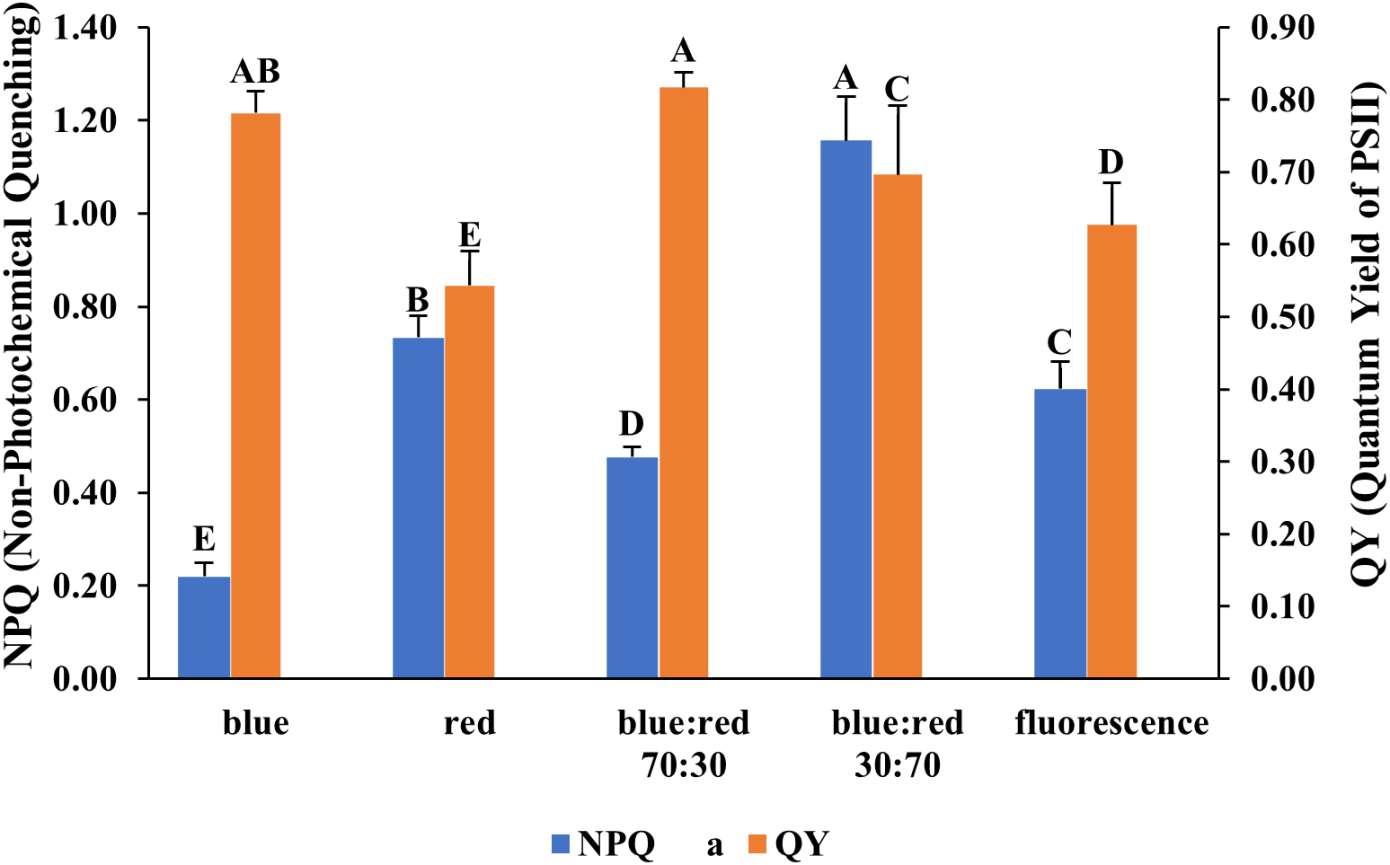
Effect of different light qualities on non-photochemical quenching (NPQ) and quantum yield of PSII (QY) in *Bucephalandra* sp. ‘Wavy Dark Green’ cultured in a TIS. Light treatments included monochromatic blue and red, fluorescent light, and mixed blue:red light at 70:30 and 30:70 ratios. NPQ and QY were measured using a FluorPen fluorometer. Bars represent means ± standard deviation (n = 5). Different uppercase letters above the bars indicate statistically significant differences among treatments based on one-way ANOVA followed by Tukey’s HSD test (p < 0.05).

**Figure 7:**
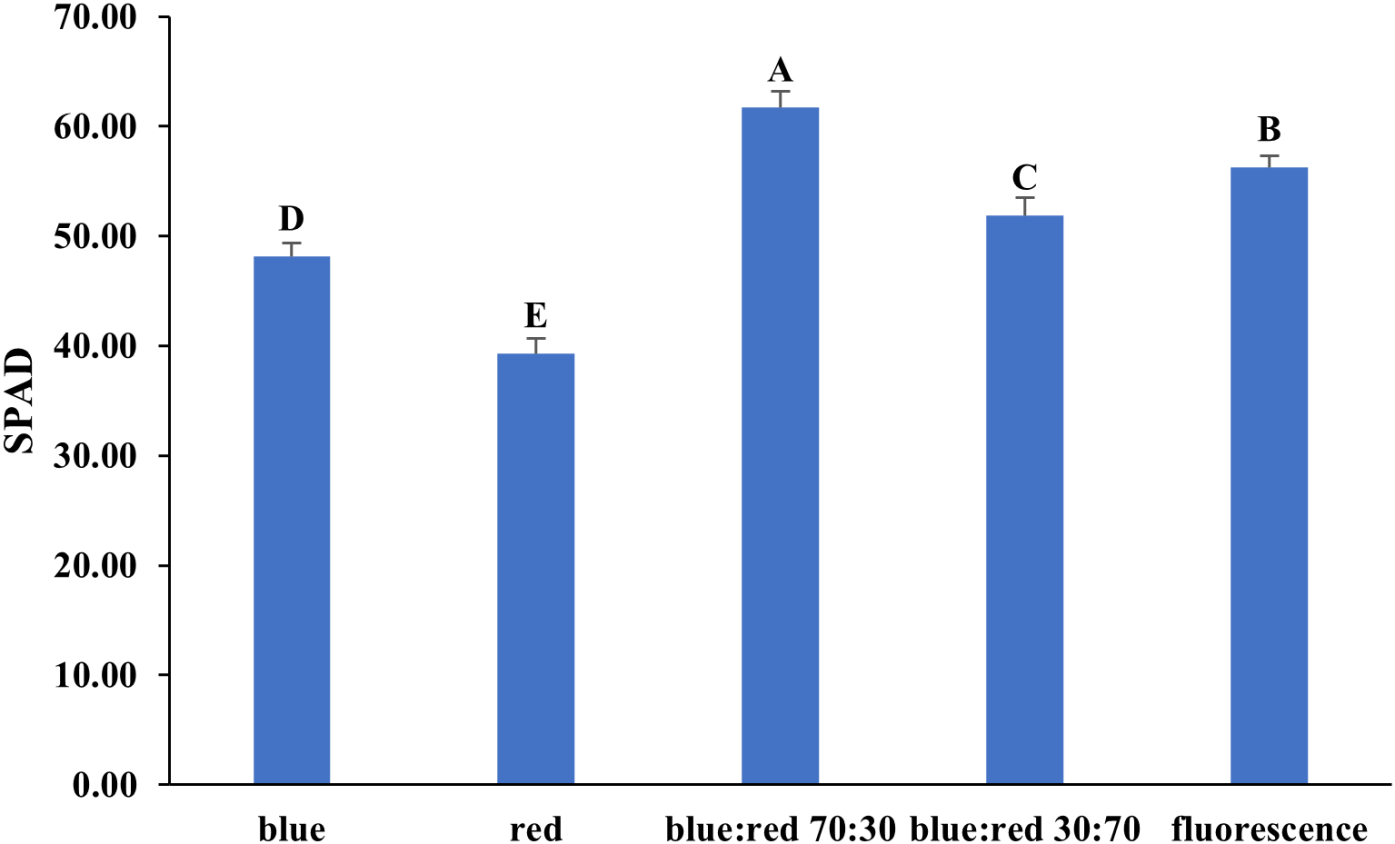
Effect of different light qualities on SPAD values in *Bucephalandra* sp. ‘Wavy Dark Green’ cultured in a temporary immersion bioreactor (TIS). Light treatments included blue, red, blue:red at 70:30 and 30:70 ratios, and fluorescent light. SPAD readings were recorded using a SPAD-502 chlorophyll meter after four weeks of culture. Bars represent means ± standard deviation (n = 5). Different uppercase letters above the bars indicate statistically significant differences among treatments (p < 0.05), as determined by one-way ANOVA followed by Tukey’s HSD test.

The effects of different light spectra on the photosynthetic performance of *Bucephalandra* sp. ‘Wavy Dark Green’ were assessed using non-photochemical quenching (NPQ) and quantum yield (QY) measurements of photosystem II. The highest QY values (~0.78–0.80), indicating efficient photochemical energy conversion, were recorded under blue light and blue:red (70:30) treatments (**Figure 6**). Conversely, the highest NPQ value (~1.15), reflecting strong photoprotective thermal dissipation, occurred under the blue:red (30:70) treatment, while the lowest NPQ (~0.22) was under blue light (**Figure 6**). SPAD values, indicative of relative chlorophyll content, also varied significantly among treatments. The highest SPAD value was recorded under blue:red (70:30), followed by fluorescent light, blue:red (30:70), blue light, and the lowest under red light (**Figure 7**).

### 3.6 Effects of light spectra on stomatal characteristics in *Bucephalandra* sp. ‘Wavy Dark Green’ cultured in TIS

The light spectra significantly influenced stomatal behavior and morphology in *Bucephalandra* sp. cultured in TIS (**Table 8**; **Supplementary Figure S5**).

**Table 8:** Effects of different light spectra on the number of open and closed stomata, stomatal length, and stomatal width in *Bucephalandra* sp. ‘Wavy Dark Green’ cultured in TIS.

Treatments	Open stomata	Closed stomata	stomatal length (µM)	stomatal width (µM)
Blue	25.00 ± 1.00a	27.33 ± 1.15b	11.83 ± 0.33a	16.16 ± 0.55a
Red	12.33 ± 1.53e	37.00 ± 2.65a	10.58 ± 0.32b	13.75 ± 0.50b
Blue: Red (70:30)	21.00 ± 1.00b	26.33 ± 2.52b	11.00 ± 0.27ab	14.66 ± 0.49ab
Blue: Red (30:70)	18.33 ± 1.53c	30.33 ± 2.08b	11.33 ± 0.38ab	14.75 ± 0.14ab
Fluorescent	15.00 ± 1.00d	30.00 ± 2.00b	11.58 ± 0.60a	15.83 ± 0.85a

Different letters within a column indicate significant differences among treatments at p < 0.05 (ANOVA, Tukey’s HSD).

#### 3.6.1 Stomatal opening and closing

Blue light most effectively promoted stomatal opening, resulting in the highest number of open stomata (25.00 ± 1.00), a value significantly greater than all other treatments (p < 0.05). The optimal growth condition, blue:red (70:30), also showed a high number of open stomata (21.00 ± 1.00). Conversely, the highest number of closed stomata (37.00 ± 2.6) was recorded under red light, which simultaneously yielded the lowest number of open stomata (12.33 ± 1.53).

#### 3.6.2 Stomatal size

Stomatal size followed a similar pattern. Blue light induced the largest stomata, resulting in both the longest (11.83 ± 0.33 µm) and widest (16.16 ± 0.55 µm) stomata, with the width being significantly greater than under red light (p < 0.05). Overall, these findings suggest that blue light and blue:red combinations are superior to red light alone in promoting stomatal opening and enlargement, which is beneficial for optimizing gas exchange and photosynthetic efficiency under *in vitro* TIS conditions.

## 4 Discussion

Surface sterilization is a critical initial step in establishing aseptic *in vitro* cultures, particularly for aquatic plants like *Bucephalandra* sp. ‘Wavy Dark Green’, which are prone to microbial contamination due to their native moist habitats. This study assessed the efficacy of several sterilization treatments using sodium hypochlorite (NaOCl) and mercuric chloride (HgCl_2_), applied either as single-step or sequential protocols. The results clearly demonstrated that two-step treatments—NaOCl followed by NaOCl or NaOCl followed by HgCl_2_—significantly improved explant survival and reduced contamination. Among the NaOCl combinations, the most effective protocols involved 0.6% NaOCl for 5 minutes followed by either 0.05% or 0.1% HgCl_2_ for 5 minutes, both achieving a survival rate of 85.71%. However, single-step HgCl_2_ treatments at 0.1–0.2% consistently achieved the highest survival with complete decontamination (100%), highlighting their superior sterilization efficacy. These findings are in agreement with previous studies on aquatic and semi-aquatic plants such as *Anubias nana* and *Rotala macrandra* (Azmi et al., 2019), *Cryptocoryne* (Dharmasiri et al., 2025), *Centella asiatica* (Divyashree et al., 2024), which confirmed the high effectiveness of HgCl_2_ in eliminating microbial contaminants without compromising tissue viability. While NaOCl is safer and more accessible, it exhibited moderate disinfection potential and required longer exposures to achieve comparable results, as similarly reported for pecan (Vahdati et al., 2020) and *Gerbera jamesonii* Bolus (Kumar et al., 2021). Furthermore, the sequential disinfection approach offers a practical alternative where the use of heavy metals like HgCl_2_ is limited due to toxicity concerns. Comparable sequential strategies have been successfully employed in the micropropagation of *Dendrobium longicornu* (Dohling et al., 2012). Overall, the dual-step NaOCl protocol and HgCl_2_ treatments at 0.1–0.2% emerged as effective and reproducible disinfection strategies for *Bucephalandra* sp. ‘Wavy Dark Green’, supporting the successful initiation of aseptic cultures and enhancing reliability in tissue culture systems.

With a reliable sterilization protocol in place, the next phase of the study examined the effects of different culture media and plant growth regulator (PGR) combinations on *in vitro* morphogenesis—a crucial step for scaling up propagation for conservation and commercial applications. The results demonstrated a strong influence of benzyladenine (BA) and naphthaleneacetic acid (NAA) concentrations on shoot and root development. Higher concentrations of BA (4–5 mg/L) significantly enhanced shoot proliferation, while low NAA levels (0.1–0.3 mg/L) supported root initiation and elongation. These findings are consistent with prior studies on aquatic and semi-aquatic species, which emphasized the central role of cytokinins in promoting axillary shoot development and the variable effects of auxins based on concentration and ratio (George et al., 2008; Rout et al., 2000). Notably, 5 mg/L BA alone produced the highest shoot and root numbers, echoing results reported for *Anubias barteri* (Giang et al., 2024; Kanchanapoom et al., 2012), *Cryptocoryne wendtii* (Kane et al., 1999), and *Bacopa monnieri* (Debnath, 2008), where shoot multiplication was maximized at moderate to high BA levels with minimal auxin input. Conversely, elevated NAA concentrations in the absence of BA suppressed shoot formation, corroborating findings by Šmeringai et al. (2023), who observed that high auxin levels can inhibit cytokinin-mediated shoot organogenesis. While BA alone did not always promote rooting, the addition of 0.3–0.5 mg/L NAA triggered adventitious root formation, aligning with earlier reports on orchids. (Reddy et al., 2020) and *Magnolia sirindhorniae* Noot. & Chalermglin (Cui et al., 2019). In contrast, excessive NAA (>0.5 mg/L) or its complete absence led to reduced rooting, indicating a biphasic, concentration-dependent response. Interestingly, cultures exposed to 5 mg/L BA without NAA produced compact, green shoots with minimal hyperhydricity—an often-cited issue in the tissue culture of most plants (Polivanova and Bedarev, 2022; Sreelekshmi and Siril, 2021). Previous studies have shown that optimal BA concentrations can mitigate vitrification by stabilizing water balance and cytokinin sensitivity (Ziv, 1991). Comparable morphological responses were also noted in *Aronia melanocarpa* (Bayhan and Yücesan, 2024) and *Tectona grandis* (Quiala et al., 2014), reinforcing the consistency of our findings. While the medium that maximized shoot production was not optimal for root formation, subsequent experiments were designed to determine more effective rooting media.

Root formation plays a vital role in the acclimatization and survival of *in vitro*–propagated plants. This study investigated the effects of varying MS medium strengths and NAA supplementation on root development in *Bucephalandra* sp. ‘Wavy Dark Green’. The results indicated that reducing the salt concentration—particularly to ¼ MS—significantly improved both root number and length compared to full-strength MS or NAA-supplemented media. This improved rooting response in ¼ MS may be due to reduced osmotic stress and ion toxicity, conditions that have been shown to promote root organogenesis in many plant species (Chae, 2014; Shekhawat et al., 2015; Tawfik et al., 2019; Waseem et al., 2009). Nutrient dilution may also enhance endogenous auxin activity, thereby facilitating efficient root primordia formation without interference from excessive salts (George et al., 2008). In contrast, the addition of NAA—especially at 0.3–0.5 mg/L—negatively affected both root number and elongation, in agreement with earlier reports showing that high auxin levels can impair cellular polarity, shorten elongation zones, and result in malformed roots (De Klerk et al., 1999). Excessive exogenous auxin may also disrupt the plant’s internal hormone equilibrium, particularly in species sensitive to auxin overdose (Zhao et al., 2022). These observations are consistent with findings in *Cistus creticus* L., where root induction improved under reduced MS salt strength and in the absence of auxin (Ioannidis and Koropouli, 2024). As such, diluted MS medium without added PGRs appears to be a preferable and cost-effective approach for rooting aquatic ornamentals like *Bucephalandra* sp. ‘Wavy Dark Green’. Avoiding unnecessary hormone supplementation not only improves morphological quality but also reduces costs and minimizes physiological disorders, which is essential for commercial-scale micropropagation.

Once a suitable rooting medium was identified, the next and equally critical step in micropropagation was the acclimatization of plantlets to *ex vitro* conditions. This phase is particularly challenging for aquatic species such as *Bucephalandra* sp. ‘Wavy Dark Green’, which require tightly regulated humidity levels to ensure successful transition. Both excessive and insufficient moisture during this period can lead to tissue rot or desiccation, underscoring the importance of selecting a substrate that supports a stable microenvironment (Hazarika, 2006; Kozai et al., 2005; Pospíšilová et al., 1999). In this study, humidity during acclimatization was carefully controlled by housing plantlets in transparent plastic containers with lids perforated by small ventilation holes. This setup allowed for a gradual reduction in relative humidity while preventing excess moisture accumulation, thereby mitigating transplant shock and improving survival rates. The superior performance of plantlets grown in sand, perlite, and vermiculite mixtures can be attributed to these substrates—high porosity and excellent drainage properties—key factors in reducing transplant stress and enhancing root oxygenation. Such conditions help maintain an optimal balance of air and water around the root zone, facilitating root establishment and water uptake (Pospíšilová et al., 1999). Previous research has shown that inert substrates like sand and perlite are particularly effective in minimizing water stress and improving acclimatization success in micropropagated species adapted to semi-aquatic or humid environments (Heintze et al., 2018; Kozai et al., 2005). Additionally, the capillary action and structural characteristics of these substrates promote gradual water movement and nutrient retention without waterlogging, thereby supporting stable growth (Debnath, 2007). In contrast, substrates such as black rice husk may retain excessive moisture, resulting in poor aeration and heightened susceptibility to microbial contamination, ultimately reducing plantlet survival (Hazarika, 2006). These results align with findings in other tropical ornamentals, including *Philodendron* and *Anthurium*, where the use of well-draining substrates significantly improved acclimatization outcomes and root development (Klanrit et al., 2023; Lan and Anh, 2018). Thus, substrate selection is a critical factor in the successful transition from *in vitro* to *ex vitro* culture and must be tailored to the physiological needs and ecological adaptations of each plant species.

This study reaffirms the species-specific nature of plant growth regulator (PGR) responses, particularly in aquatic taxa, where anatomical adaptations to water influence hormone perception, distribution, and transport (Lloyd and McCown, 1980). The combination of 5 mg/L BA and the absence of NAA was identified as optimal for direct shoot induction in *Bucephalandra* sp. ‘Wavy Dark Green’ cultured on conventional semi-solid media. However, the inherently slow growth rate and limited shoot proliferation characteristic of *Bucephalandra* highlight the limitations of traditional *in vitro* techniques for large-scale propagation (Murthy et al., 2023; Paek et al., 2005). To overcome these constraints, a temporary immersion system (TIS) was implemented as an alternative culture strategy. TIS has been widely recognized for enhancing micropropagation efficiency in various plant species by improving nutrient uptake, reducing hyperhydricity, and supporting superior shoot quality through periodic liquid-phase exposure (Etienne and Berthouly, 2002; Georgiev et al., 2014). In the present study, a regime of six immersions per day, each lasting two minutes, was determined to be optimal. This frequency provided a favorable balance between nutrient availability and gaseous exchange, resulting in improved shoot multiplication and morphological consistency. The integration of optimized PGR regimes with TIS thus represents a robust and scalable platform for the propagation of *Bucephalandra* sp. ‘Wavy Dark Green’. This approach offers practical advantages for *ex situ* conservation, commercial aquascaping, and the export-oriented ornamental plant industry (Alister et al., 2005; Loyola-Vargas and Ochoa-Alejo, 2018).

Frequent immersions, as demonstrated in this study, enhanced shoot multiplication and fresh weight accumulation, corroborating earlier findings in *Gerbera jamesonii* (Lim et al., 2024). These results underscore the importance of frequent nutrient supply in stimulating cytokinin-responsive pathways that regulate shoot induction and elongation (Etienne and Berthouly, 2002). They also support the hypothesis that regular nutrient renewal maintains optimal nutrient and oxygen availability at the explant surface, thereby promoting shoot initiation and overall growth (Etienne and Berthouly, 2002; Georgiev et al., 2014). However, while beneficial for shoot development, frequent immersions may inhibit root formation, potentially due to excessive tissue hydration or localized hypoxic conditions (Etienne and Berthouly, 2002; Steinmacher et al., 2011).

Immersion duration proved equally critical to shoot development in TIS. Prolonged immersion times (beyond 2 minutes) led to diminished growth, likely due to reduced oxygen diffusion and the onset of hyperhydricity, as reported in *Ficus religiosa* (Polivanova and Bedarev, 2022) and *Rosmarinus officinalis* L (Villegas-Sánchez et al., 2021). These outcomes are consistent with the hypothesis that oxygen limitation during immersion impairs mitochondrial respiration, thereby reducing the energy available for cell division and organogenesis (Ziv, 2005). More broadly, these findings emphasize the need to tailor TIS protocols to the physiological and anatomical traits of each species. For instance, aquatic and semi-aquatic plants such as *Ludwigia palustris* (L.) Ell exhibit species-specific responses to immersion parameters due to their inherent water-related adaptations (Fontanili et al., 2014). In this study, minimal hyperhydricity was observed under the 2-minute, six-times-daily immersion regime, consistent with previous reports highlighting TIS’s potential to mitigate vitrification—an often-limiting factor in liquid culture systems (Berthouly and Etienne, 2005; Carvalho et al., 2019). These findings validate TIS as a suitable and scalable system for the propagation of *Bucephalandra* sp. ‘Wavy Dark Green’, especially where shoot integrity and post-acclimatization survival are crucial. The identified optimal immersion regime significantly enhanced shoot proliferation, supporting commercial propagation and *ex situ* conservation goals. While immersion parameters are key, this study also demonstrated that light quality plays a pivotal role in regulating morphogenic outcomes in TIS-grown plantlets. In particular, blue-enriched LED lighting significantly influenced shoot elongation, chlorophyll content, and stomatal development in *Bucephalandra* sp. ‘Wavy Dark Green’, corroborating previous findings in many slow-growing species (Hernández and Kubota, 2016; Hogewoning et al., 2010; Li et al., 2020). Integrating optimized light spectra with immersion control thus represents a synergistic strategy for maximizing propagation efficiency and plantlet quality. These observations further support studies emphasizing the combined effects of blue and red light on tissue culture growth and development (Gupta and Jatothu, 2013; Muneer et al., 2014). Blue wavelengths appear to promote cell division and elongation (Hogewoning et al., 2010; Kong and Zheng, 2024), whereas red light alone was insufficient for robust shoot development (Nhut et al., 2003). Fluorescent light, despite higher energy demands than LEDs, also stimulated shoot and leaf growth, consistent with previous report (Lin et al., 2013). The superior response under the blue:red (70:30) spectrum highlights the importance of spectral balance in light quality optimization. Blue-dominant light likely enhances chlorophyll biosynthesis and photosynthetic activity, promoting biomass accumulation and morphogenesis (Heo et al., 2002; Li and Kubota, 2009). Similar findings have been reported in lettuce (*Lactuca sativa* L) (Bula et al., 1991), carnation (Manivannan et al., 2017), and *Cymbidium* (Tanaka et al., 1998), where light quality modulated shoot proliferation and physiological behavior, these results underscore the necessity of species-specific light optimization to achieve high-efficiency micropropagation outcomes in aquatic ornamental plants.

To further elucidate the physiological mechanisms underlying the observed growth responses of *Bucephalandra* sp. ‘Wavy Dark Green’ cultured in TIS under varying light spectra, transcriptional profiling of key light-responsive genes was conducted. While the optimized TIS regime (six immersions per day for two minutes) in combination with specific light treatments enhanced shoot proliferation and chlorophyll accumulation, the molecular basis of these responses remained unclear. Therefore, gene expression analysis targeting photosynthesis- and energy metabolism-related genes provided insight into how spectral composition modulates cellular processes in aquatic ornamentals. Among the tested conditions, the blue:red (70:30) LED treatment consistently induced the highest expression levels of all assessed genes—particularly *petG*, *psbB*, *pskB*, and *atpA*—indicating enhanced activity of the cytochrome b6f complex and ATP synthase under this spectral balance. These findings are consistent with previous studies showing that blue-light-enriched spectra stimulate transcription of photosystem-related genes, thereby improving photochemical efficiency and biomass accumulation in diverse plant species (Hogewoning et al., 2010; Johkan et al., 2010; Li and Kubota, 2009). Notably, *petG*, which encodes a subunit of the cytochrome b6f complex essential for electron transport between PSII and PSI, showed particularly strong upregulation under the blue:red treatment. Similar trends have been reported in crops such as lettuce (Muneer et al., 2014) and tomato (Arif et al., 2024) underscoring the general role of blue-red light synergy in enhancing photosynthetic electron transport. The concurrent upregulation of *atpA* and *pskB* suggests increased ATP synthesis and photosystem II activity, processes that are critical for maintaining energy balance and supporting carbon fixation (Chen et al., 2004; Matsuda et al., 2004). Interestingly, while blue light alone has been associated with enhanced stomatal conductance and pigment biosynthesis (Hernández and Kubota, 2016; Massa et al., 2008), its sole application in this study led to lower expression of photosynthetic genes. This divergence may reflect species-specific light perception and the interaction of spectral quality with anatomical and physiological traits unique to aquatic plants (Chibani et al., 2025; Liu and van Iersel, 2021). In contrast, fluorescent light induced the lowest expression across all target genes, reinforcing previous reports of its limited efficiency in modulating transcriptional responses relative to LEDs (Ren et al., 2018; Seiler et al., 2017). The expression patterns observed in this study suggest that mixed-spectrum LEDs, particularly those enriched in blue light, more closely resemble the spectral conditions found in underwater environments—conditions to which *Bucephalandra* is naturally adapted (Liu and van Iersel, 2021). This spectral mimicry may activate photoreceptor-mediated signaling pathways that regulate plastid development and the expression of nuclear-encoded photosynthetic genes (Chen et al., 2004; Sager and McFarlane, 1997). Collectively, these findings support the strategic use of optimized light quality not only to enhance morphological parameters but also to induce favorable transcriptional reprogramming of photosynthetic machinery. This dual benefit strengthens the case for spectral optimization as a core component in the micropropagation of aquatic ornamental species.

The observed variations in NPQ (non-photochemical quenching) and QY (quantum yield) across different light treatments underscore the critical role of spectral quality in modulating photosynthetic performance in *Bucephalandra* sp. ‘Wavy Dark Green’ cultured in TIS. High NPQ values, particularly under the blue:red (30:70) treatment, suggest an enhanced thermal dissipation capacity, likely functioning as a photoprotective mechanism against excess light energy (Demmig-Adams and Adams, 1992; Müller et al., 2001). This response is typically associated with the activation of the xanthophyll cycle and the upregulation of energy-dissipating pathways in photosystem II (Niyogi, 1999). In contrast, blue light treatments resulted in the highest QY and the lowest NPQ, indicating a more efficient conversion of absorbed light into photochemical energy with minimal heat dissipation. Blue wavelengths are known to stimulate photochemical quenching and promote the development of photosynthetically active structures, contributing to greater photosynthetic efficiency (Hogewoning et al., 2010; Johkan et al., 2010). The dual peak in QY observed under both pure blue light and the blue:red (70:30) combination supports previous findings that these spectra promote optimal energy utilization and balanced photosynthetic activity (Li and Kubota, 2009). Fluorescent lighting yielded intermediate values for both QY and NPQ. This may be attributed to its broad-spectrum output, which provides moderate activation across multiple photoreceptors but lacks the spectral specificity and efficiency of LED systems (Lin et al., 2013). Red light alone produced lower QY and moderately high NPQ, consistent with reports that monochromatic red light can induce photosystem imbalances or photoinhibition when not complemented by blue wavelengths (Muneer et al., 2014). These patterns are in agreement with findings from other species such as *Brosimum gaudichaudii*, marigold, salvia, and lettuce, where blue:red combinations—especially blue-enriched treatments—favored high QY and efficient energy partitioning (Costa et al., 2021; Heo et al., 2002; Yudina et al., 2022). Moreover, the elevated NPQ observed under the blue:red (30:70) regime suggests activation of stress-adaptive photoprotection, a response particularly relevant under the high-light conditions typical of TIS environments (Massa et al., 2008). Overall, these findings reinforce the importance of optimizing light spectral composition to enhance photosynthetic efficiency while mitigating photooxidative stress in *in vitro* propagation systems for aquatic ornamentals.

Light quality is a critical determinant of photosynthetic pigment accumulation in *in vitro*-cultured plants. In this study, *Bucephalandra* sp. ‘Wavy Dark Green’ exhibited significant variation in SPAD values under different spectral compositions, reflecting differences in chlorophyll content and, by extension, photosynthetic potential. The highest SPAD value was recorded under the blue:red (70:30) LED treatment, followed by fluorescent light, blue:red (30:70), pure blue, and red light, respectively. These results suggest that a higher proportion of blue light, when properly balanced with red, promotes chlorophyll biosynthesis in aquatic species. Blue light is known to play a pivotal role in stomatal development and the regulation of genes involved in chlorophyll synthesis. However, combinations of blue and red wavelengths typically outperform monochromatic treatments, as they simultaneously activate both cryptochromes and phytochromes. This dual activation enhances chloroplast development and promotes pigment accumulation (Li and Kubota, 2009; Miao et al., 2015). The superior performance of the 70:30 blue:red ratio observed in this study aligns with prior findings in spinach, radish, and lettuce (Yorio et al., 2001) and strawberry (Nhut et al., 2003), where similar spectral balances maximized chlorophyll concentration and biomass accumulation. The lowest SPAD value under red light alone highlights its limited efficacy in promoting chlorophyll biosynthesis in the absence of blue wavelengths. This outcome is consistent with observations in other shade-adapted and aquatic species, where blue light has been shown to be essential for photomorphogenic development and pigment production (Marques et al., 2021; Muneer et al., 2014). Fluorescent lighting, despite being less energy-efficient than LEDs, produced relatively high SPAD values due to its broad spectral output, reinforcing the importance of spectral diversity for effective pigment synthesis. Collectively, these findings emphasize that spectral quality—particularly the optimal blue:red ratio—is critical for maximizing photosynthetic pigment accumulation and ensuring vigorous plantlet development in aquatic ornamentals such as *Bucephalandra* sp. ‘Wavy Dark Green’. Cultivation under blue:red (70:30) conditions may therefore offer a practical and effective lighting strategy for both *in vitro* and ex vitro propagation systems.

Light quality significantly influences stomatal development, density, and aperture regulation, all of which are critical for optimizing gas exchange and photosynthetic efficiency. In this study, *Bucephalandra* sp. ‘Wavy Dark Green’ cultured under blue light exhibited the highest number of open stomata, along with increased stomatal length and width. These observations suggest that blue wavelengths strongly promote both stomatal development and opening. This is consistent with previous studies indicating that blue light activates phototropins and stimulates plasma membrane H^+^-ATPase activity in guard cells, thereby facilitating stomatal opening (Inoue and Kinoshita, 2017; Shimazaki et al., 2007). In contrast, red-light treatment resulted in the lowest number of open stomata and the highest number of closed stomata, aligning with reports that red light alone is insufficient to trigger full stomatal opening due to the lack of blue light receptor activation (Boccalandro et al., 2009; Talbott and Zeiger, 1993). This limited effect is largely attributed to red light’s reliance on phytochrome signaling pathways, which do not directly activate the mechanisms necessary for guard cell turgor changes. Combined blue:red treatments, particularly at a 70:30 ratio, produced intermediate but significantly enhanced responses, including a higher proportion of open stomata and enlarged stomatal dimensions. These findings support previous studies demonstrating synergistic effects of red and blue light on stomatal physiology and leaf development (Hogewoning et al., 2010; Ouzounis et al., 2015). The blue-enriched spectra appear to optimize stomatal opening while red wavelengths may contribute to overall photosynthetic balance. Fluorescent lighting, which emits a broad spectrum including both blue and red wavelengths, promoted moderate stomatal opening and development. However, its effects were less pronounced compared to pure blue light, indicating that the specific spectral composition—rather than light intensity alone—is the key determinant of stomatal response (Hernández and Kubota, 2016). The ability of blue light to enhance stomatal aperture has been widely exploited in controlled environment agriculture to increase CO_2_ uptake efficiency and biomass production. In conclusion, these findings confirm that spectral quality—especially the presence and dominance of blue wavelengths—plays a pivotal role in modulating stomatal development and aperture regulation in *Bucephalandra* sp. ‘Wavy Dark Green’. Optimizing these light parameters *in vitro* can therefore improve photosynthetic performance and water-use efficiency in aquatic ornamental species.

While the optimized TIS immersion regime and blue:red LED spectrum yielded strong *in vitro* responses, several limitations should be acknowledged. Responses may differ among *Bucephalandra* species, large-scale TIS operation introduces technical challenges related to contamination control, cost of LED installation, and the need for skilled personnel. The acclimatization stage also remains a major bottleneck, as plantlets are highly sensitive to humidity and substrate conditions. Furthermore, the economic viability of maintaining species-specific light spectra in commercial nurseries requires evaluation. These factors highlight the importance of species validation and techno-economic assessment prior to commercial adoption.

## 5 Conclusion

This study establishes an improved and efficient micropropagation for *Bucephalandra* sp. ‘Wavy Dark Green’ by integrating critical parameters including explant sterilization, plant growth regulator (PGR) optimization, temporary immersion system (TIS) immersion regimes, and light spectral modulation. Results indicate that a TIS regime with six immersions per day for 2–3 minutes, in combination with Murashige and Skoog (MS) medium supplemented with 5 mg/L BA and a blue:red (70:30) LED lighting ratio, significantly enhanced shoot proliferation, chlorophyll accumulation, and the expression of genes associated with photosynthesis and energy metabolism. Root induction was effectively achieved using low concentrations of NAA, while acclimatization in well-drained sand substrates resulted in high survival rates. The study provides novel insights into light-regulated gene expression in aquatic ornamental species and supports the application of blue:red LED systems in advanced *in vitro* platforms such as TIS. The developed protocol offers a scalable and environmentally sustainable strategy for the commercial propagation and international distribution of *Bucephalandra* sp. ‘Wavy Dark Green’, contributing both to biodiversity conservation and to the expansion of the global ornamental plant industry.

## Data Availability

The original contributions presented in the study are included in the article/**Supplementary Material**. Further inquiries can be directed to the corresponding author.
