# Supplementary material for: Light spectrum effects on micropropagation and gene expression of Bucephalandra sp. in a temporary immersion system for sustainable production
Source: Front Plant Sci. 2025 Dec 2;16:1660632. doi: 10.3389/fpls.2025.1660632 (PMC12707052; doi:10.3389/fpls.2025.1660632)
Supplement: Supplementary file 1 [file DataSheet1.docx]

**
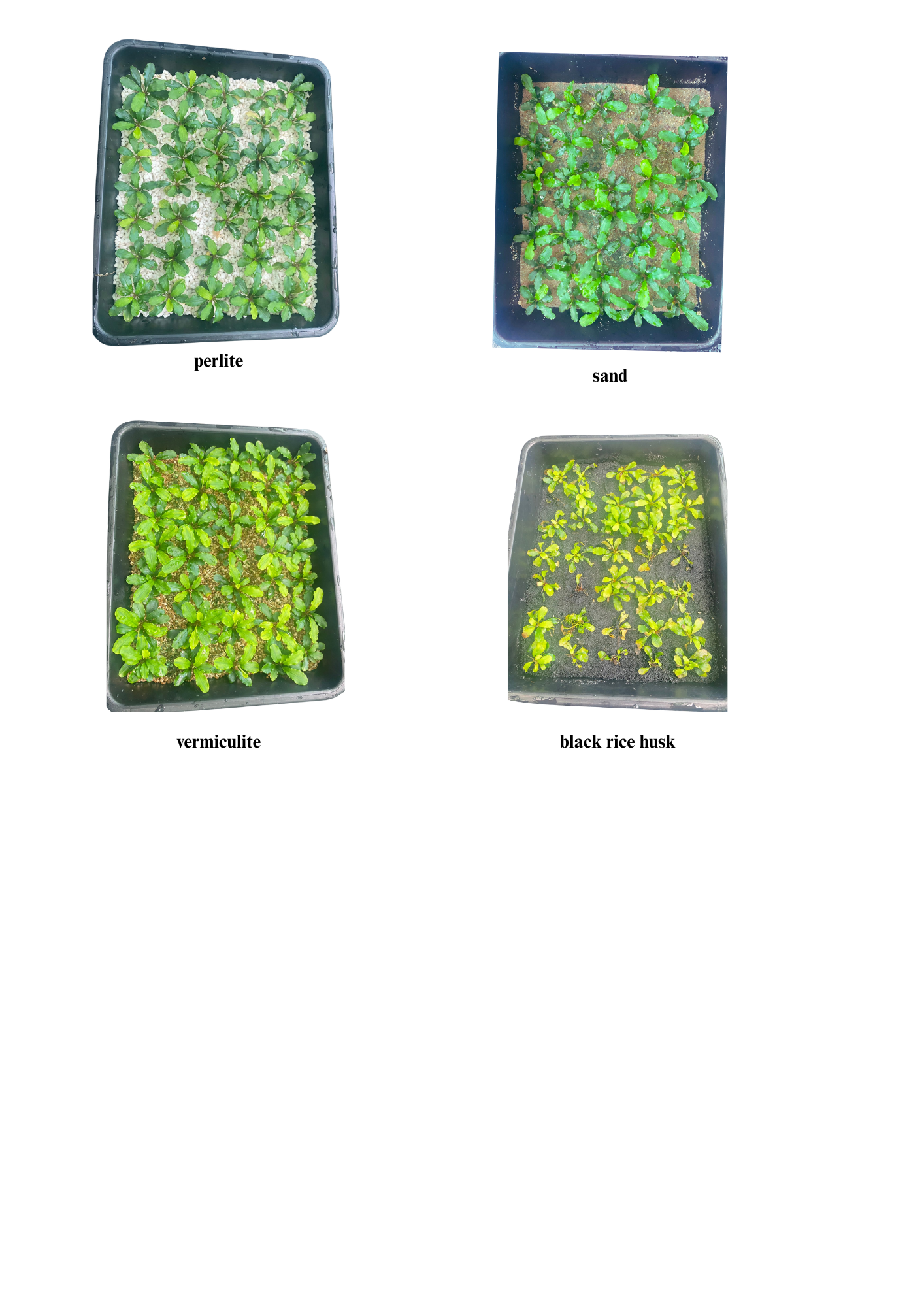
**

**Supplementary Figure S1:** Acclimatization of *Bucephalandra* sp. 'Wavy Dark Green' plantlets in different substrates, including vermiculite and sand. Plantlets grown in sand exhibited superior survival rate and overall growth vigor, indicating its favorable aeration and drainage properties compared to vermiculite-based treatments.
