# Supplementary material for: Light spectrum effects on micropropagation and gene expression of Bucephalandra sp. in a temporary immersion system for sustainable production
Source: Front Plant Sci. 2025 Dec 2;16:1660632. doi: 10.3389/fpls.2025.1660632 (PMC12707052; doi:10.3389/fpls.2025.1660632)
Supplement: Supplementary file 2 [file DataSheet2.docx]

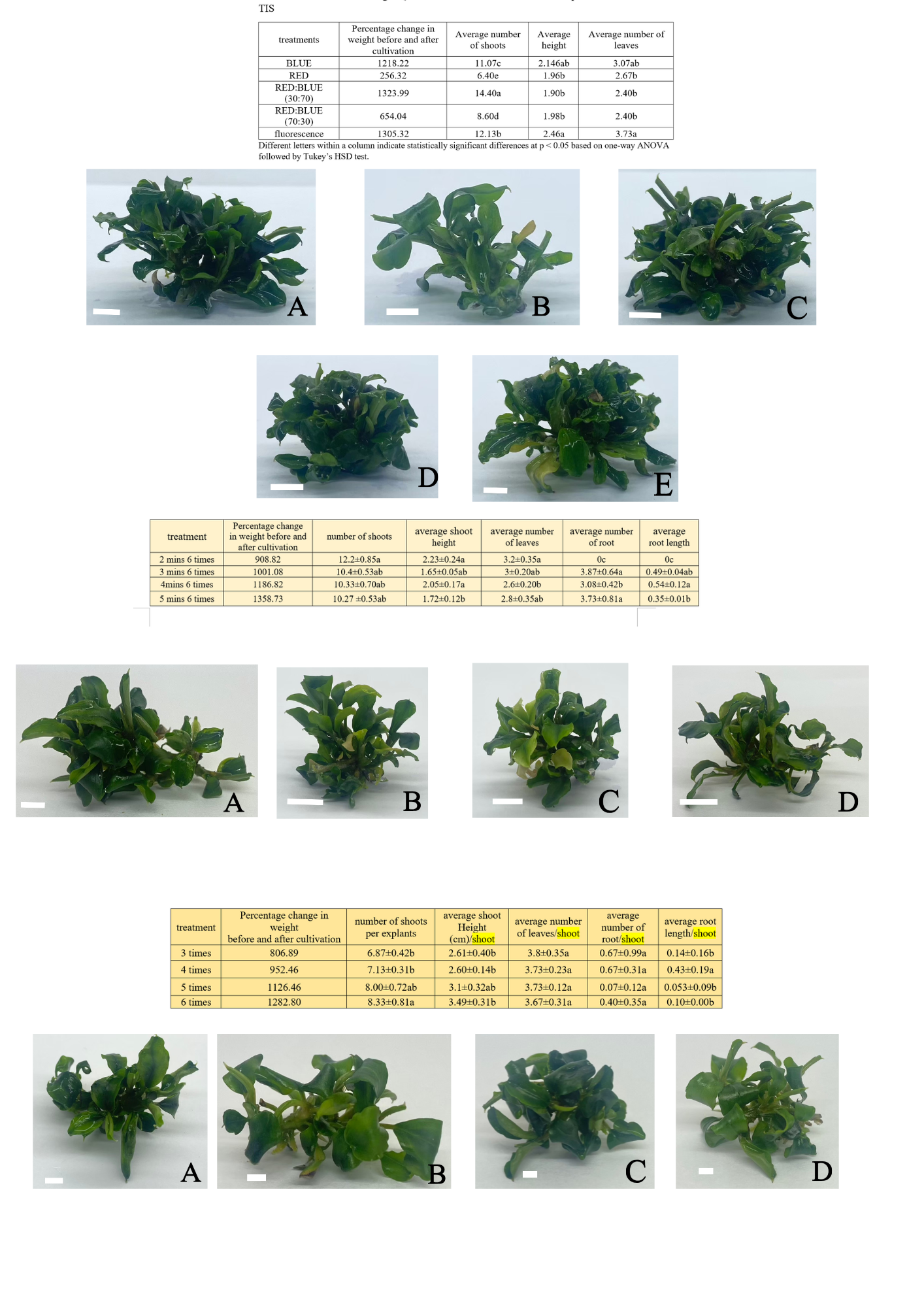


**Supplementary Figure S2:** Growth response in a TIS with immersion frequency of 3 (A), 4 (B), 5 (C), 6 (D) times per day. White bar represents 1 cm.
