# Supplementary material for: Light spectrum effects on micropropagation and gene expression of Bucephalandra sp. in a temporary immersion system for sustainable production
Source: Front Plant Sci. 2025 Dec 2;16:1660632. doi: 10.3389/fpls.2025.1660632 (PMC12707052; doi:10.3389/fpls.2025.1660632)
Supplement: Supplementary file 3 [file DataSheet3.docx]

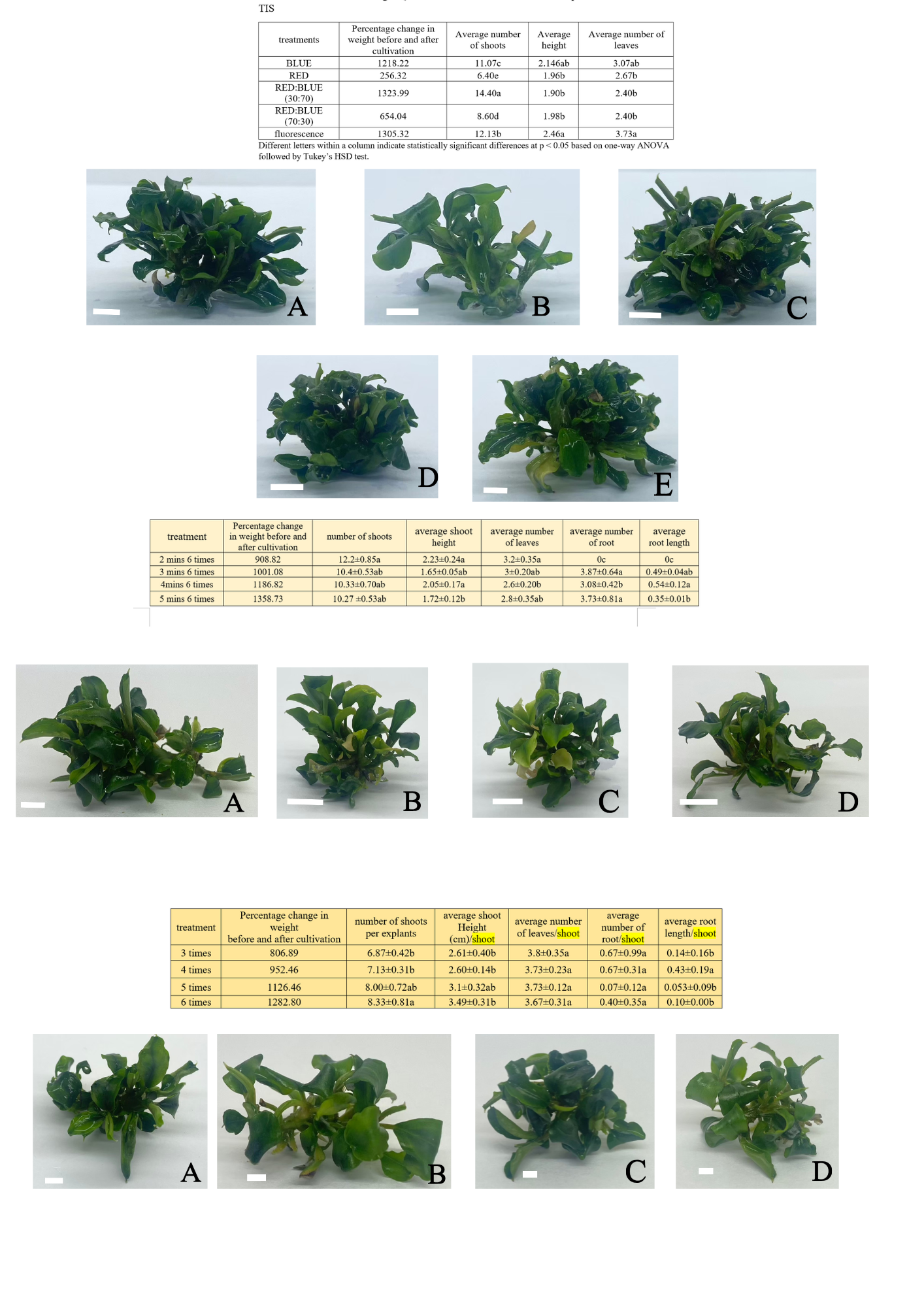


**Supplementary Figure S3:** Growth response in a TIS with an immersion frequency of 6 times per day combined with four different immersion durations: (A) 2 minutes, (B) 3 minutes, (C) 4 minutes, and (D) 5 minutes. White bar represents 1 cm.
