# Supplementary material for: Light spectrum effects on micropropagation and gene expression of Bucephalandra sp. in a temporary immersion system for sustainable production
Source: Front Plant Sci. 2025 Dec 2;16:1660632. doi: 10.3389/fpls.2025.1660632 (PMC12707052; doi:10.3389/fpls.2025.1660632)
Supplement: Supplementary file 4 [file DataSheet4.docx]

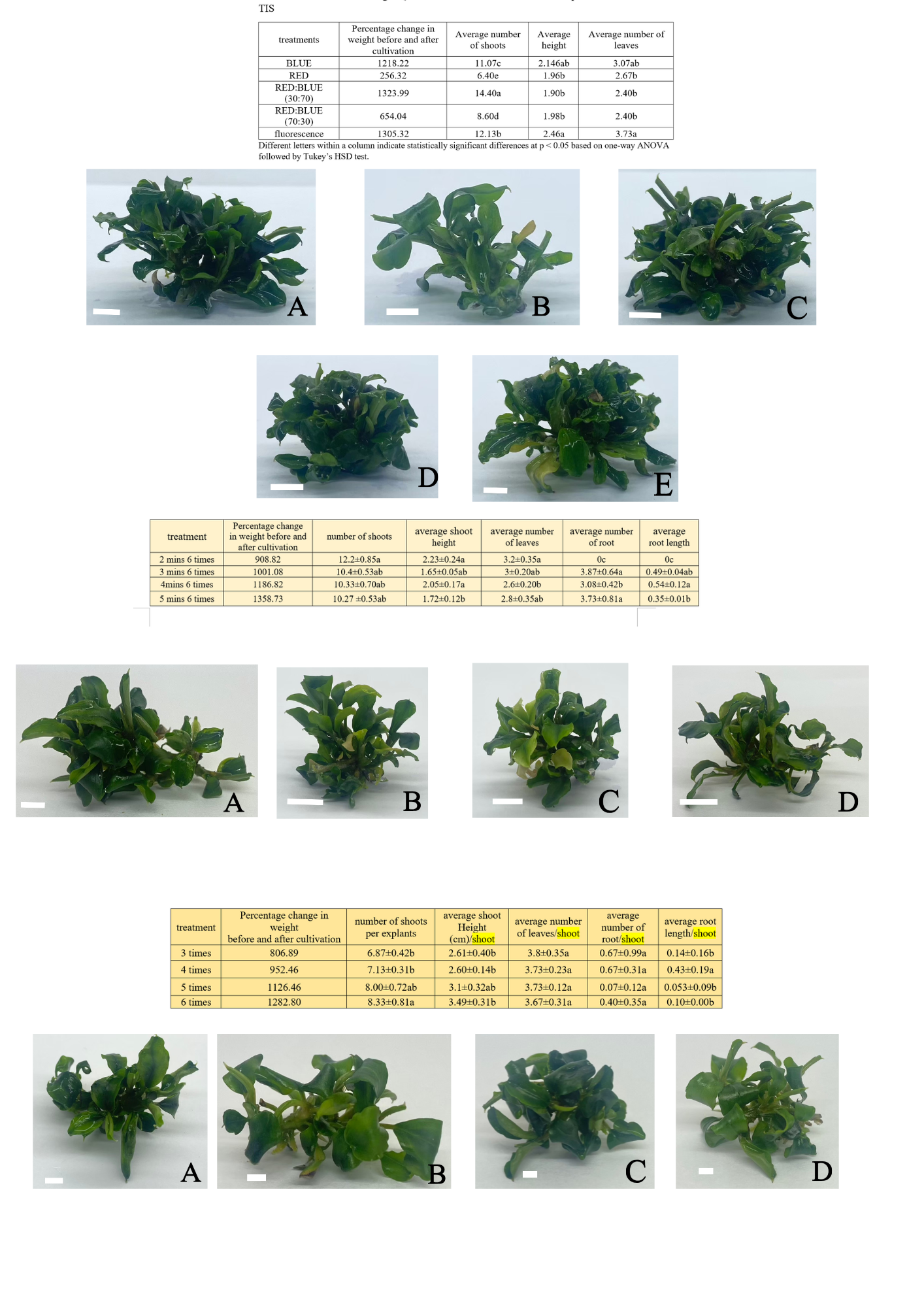


**Supplementary Figure S4** Growth response of *Bucephalandra* sp. 'Wavy Dark Green' cultured in a TIS with an immersion duration of 2 minutes, 6 times per day, under (A) blue (B) red (C) blue:red (70:30) D blue:red (30:70), and (E) fluorescent light spectrum. White bar represents 1 cm.
