# Supplementary material for: Light spectrum effects on micropropagation and gene expression of Bucephalandra sp. in a temporary immersion system for sustainable production
Source: Front Plant Sci. 2025 Dec 2;16:1660632. doi: 10.3389/fpls.2025.1660632 (PMC12707052; doi:10.3389/fpls.2025.1660632)
Supplement: Supplementary file 5 [file DataSheet5.docx]

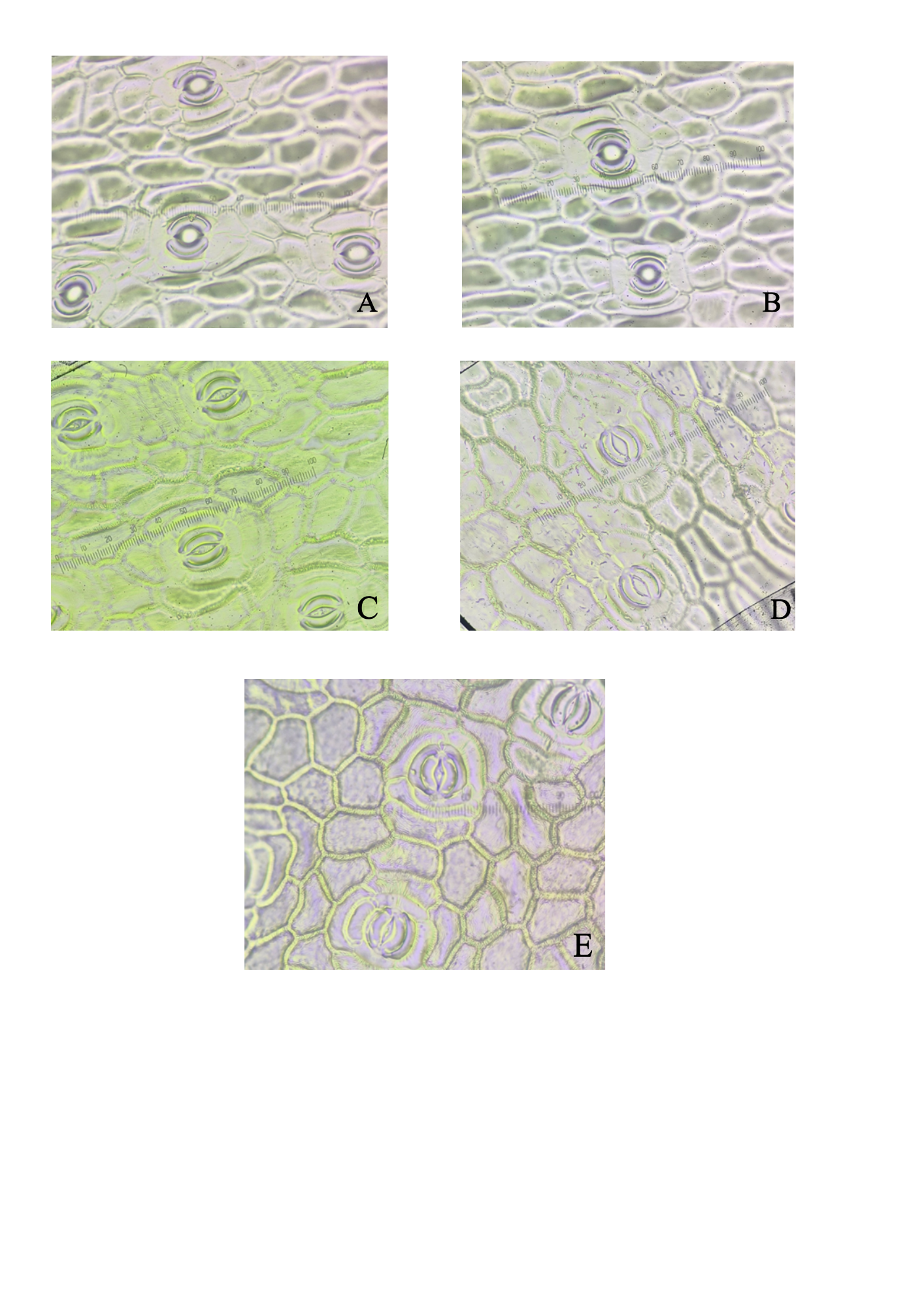


**Supplementary Figure S5** Leaf stomata (400X) in the lower epidermis of *Bucephalandra* sp. 'Wavy Dark Green' subjected to different light regime. (A) blue light, (B) blue:red 70:30, (C) blue:red 30:70, D fluorescence, and (E) red light
